# Population structure and genome-wide association studies in bread wheat for phosphorus efficiency traits using 35 K Wheat Breeder’s Affymetrix array

**DOI:** 10.1038/s41598-021-87182-2

**Published:** 2021-04-07

**Authors:** Preman R. Soumya, Amanda J. Burridge, Nisha Singh, Ritu Batra, Renu Pandey, Sanjay Kalia, Vandana Rai, Keith J. Edwards

**Affiliations:** 1 Mineral Nutrition Laboratory, Division of Plant Physiology, ICAR-Indian Agricultural Research Institute, New Delhi, 110 012 India; 2 Life Sciences, University of Bristol, 24 Tyndall Avenue, Bristol, BS8 1TQ UK; 3 ICAR-National Institute for Plant Biotechnology, Pusa Campus, New Delhi, 110 012 India; 4 Department of Biotechnology, C.G.O Complex, Lodhi Road, New Delhi, 110003 India; 5 Present Address: Regional Agricultural Research Station, Kerala Agricultural University, Ambalavayal, Wayanad, 673593 Kerala India

**Keywords:** Plant biotechnology, Plant physiology, Plant sciences, Abiotic

## Abstract

Soil bioavailability of phosphorus (P) is a major concern for crop productivity worldwide. As phosphatic fertilizers are a non-renewable resource associated with economic and environmental issues so, the sustainable option is to develop P use efficient crop varieties. We phenotyped 82 diverse wheat (*Triticum aestivum* L.) accessions in soil and hydroponics at low and sufficient P. To identify the genic regions for P efficiency traits, the accessions were genotyped using the 35 K-SNP array and genome-wide association study (GWAS) was performed. The high-quality SNPs across the genomes were evenly distributed with polymorphic information content values varying between 0.090 and 0.375. Structure analysis revealed three subpopulations (C1, C2, C3) and the phenotypic responses of these subpopulations were assessed for P efficiency traits. The C2 subpopulation showed the highest genetic variance and heritability values for numerous agronomically important traits as well as strong correlation under both P levels in soil and hydroponics. GWAS revealed 78 marker-trait associations (MTAs) but only 35 MTAs passed Bonferroni Correction. A total of 297 candidate genes were identified for these MTAs and their annotation suggested their involvement in several biological process. Out of 35, nine (9) MTAs were controlling polygenic trait (two controlling four traits, one controlling three traits and six controlling two traits). These multi-trait MTAs (each controlling two or more than two correlated traits) could be utilized for improving bread wheat to tolerate low P stress through marker-assisted selection (MAS).

## Introduction

Wheat (*Triticum aestivum* L.) is a major staple food crop world over. To meet the needs of an increasing population, agriculturists are facing the challenge of enhancing wheat production up to 70% by 2050^1^. Increasing demand and the challenges of global climate change requires an urgent need for genetic improvement of wheat to attain maximum yield, adaptation and tolerance to various abiotic and biotic stresses. Bioavailability of phosphorus (P) is a major constraint in the wheat production system due to slow diffusion and high P-fixation in different soils. In acidic soils, P is fixed with Al and Fe oxides and hydroxides whereas in alkaline soils, it is precipitated as calcium phosphates thereby, limiting P availability and crop productivity^2^. Higher levels of P fertilization, a non-renewable resource, increase the chances of environmental pollution through eutrophication of water bodies^3^ as well as an economic burden on the resource poor agricultural sector in several countries^4^. Low bioavailability of P and less fertilizer recovery (> 30%) thus justifies the need to develop crop varieties that can acquire P more efficiently.

In plants, inorganic P (Pi) is a key structural component of bio-membranes and nucleic acids. It has role in energy transfer reactions, signalling and regulation of carbon metabolism. In response to Pi starvation, plants possess several adaptive strategies at morphological, physiological, biochemical, genetic and genomic levels. Below ground traits such as increased root surface area, total root length, increased root-to-shoot ratio, root hair length and density, along with increased exudation of organic acids, protons and Pi-solubilizing enzymes from root enhances P-mining ability^5–8^. At a molecular level, several Pi starvation-induced (PSI) genes and gene products provide tolerance to plants to cope with the P stress condition as shown by transcriptomic, proteomic and metabolomic studies^9–11^. Further, genotypic differences in P stress tolerance have been reported in many crops, for example, in soybean ^7^, maize ^11^, wheat ^12^ and rice ^13^. To improve P use efficiency (PUE) through genetic approaches, identification of a few quantitative trait loci (QTLs) have been reported which are mainly based on the shoot P concentration and biomass production^14–16^. However, the traits governing PUE needs to be defined appropriately, correct phenotyping techniques and large number of population or diverse germplasm are some of the challenges faced by breeders. Screening for P efficiency includes a comparison in shoot biomass and grain yield^17^ at low and sufficient P levels. So, the phenotypic variability can be determined through relative performance such as relative biomass and relative grain yield. Apart from phenotypic variability, information about the amount and distribution of genetic diversity provides opportunity for genetic improvement based on exploitation of genetic resources that can be used in future plant-breeding programs. In wheat, mapping of QTLs for P uptake and utilization efficiency detected six and seven loci controlling P utilization and P uptake efficiency, respectively^14,18^. It was suggested that although P uptake and utilization efficiencies are negatively correlated, the presence of positive linkages between QTLs for P uptake and utilization at two loci would allow the possibility of improving both uptake and utilization efficiencies.

Wheat is an allohexaploid with three genomes (2n = 6 ×  = 42, AABBDD), derived from three ancestral parental diploid species: *Triticum urartu* (AA), a progenitor related to *Aegilops speltoides* (BB), and *Aegilops tauschii* (DD). Large size (17 Gb) and complexity of genomes has hindered genomic analyses for elucidating the genetic diversity of wheat. However, with the sequencing of reference wheat genome, these challenges have been met. The genetic diversity may be determined by using pedigree data, morphological^19^ as well as molecular marker information^20^. Analysis of genetic diversity using morphological information is expensive, labour intensive, and may be affected by genotype and environment (G × E) interactions. Hence, deoxyribonucleic acid (DNA) markers have been used extensively to evaluate the population structure and genetic diversity of accessions^21,22^. The DNA markers such as amplified fragment length polymorphism (AFLP), restriction fragment length polymorphism (RFLP), microsatellites, and single nucleotide polymorphisms (SNPs) have been exploited to analyze the relationships and genetic diversity levels in wheat accessions^23–25^. Among these markers, SNPs are the most widely distributed sequence variations in plant genomes and are the most frequent type of sequence variation^26^. The SNP markers are highly fitted for genome-wide studies such as association mapping to detect the relationship between phenotypic variation and genetic polymorphisms^27^, population structure analysis^28^ and genomic selection. Development of SNP array was major improvement in wheat genotyping which permitted acquisition of exhaustive genotypic data in wheat^29^. The SNP array is a high-throughput and cost-efficient genotyping assay and is a robust genomic approach for elucidation of genetic variation on a genome-wide genetic diversity analysis of plants^30^.

In order to dissect the genetic basis of complex traits such as P efficiency, genome-wide association study (GWAS) based on linkage disequilibrium (LD) is employed. GWAS is a high resolution and cost-effective method that associates variation across the entire genome with phenotypes by utilizing the benefits of a large number of marker polymorphisms^31^. However, GWAS is usually carried out on a large number of populations, say more than 300. But in cases where the numbers are limited (< 100), there is still a possibility to associate the genotypically diverse and phenotypically superior lines to develop new cultivars with desirable traits for abiotic stress tolerance. Several GWAS studies have been reported on population sizes between 60 and 150^32–34^. Genome-wide analysis studies on elucidating the complex traits of P efficiency in rice ^35^, maize ^36^ and soybean^37,38^ have been reported but literature on bread wheat is limited.

In the present study, we used 82 wheat accessions to determine the phenotypic variability for low P stress tolerance and the SNP array data for studying genetic diversity, population structure and marker-trait associations (MTAs). The aim of study was: (i) to describe the genome distribution of the SNPs and their information content based on the 35 K Wheat Breeders Affymetrix-SNP array, (ii) to analyze the population structure, estimate genetic diversity and marker-trait association using SNP markers, and (iii) to identify the most diverse and P efficient genotype(s) based on the phenotypic cluster associated with the diverse population group.

## Materials and methods

### Phenotyping in soil

A set of 82 bread wheat (*Triticum aestivum* L.) accessions procured indigenously as well as from Australian Winter Cereals Collection (Australia) and CIMMYT (Mexico) were used in this study. The collection comprised of 77 cultivars and 5 landraces (Supplementary Table S1). The soil screen was carried out under natural condition at Indian Agricultural Research Institute, New Delhi, India located at 28.08°N and 77.12°E and 228.61 m above mean sea level. Phenotyping for P efficiency in soil was carried out for two consecutive seasons (2015–16 and 2016–17) at low and sufficient P levels. The Olsen P^39^ in low and sufficient P treatment soil were 2.67 and 42.2 mg P kg^−1^ soil respectively. The soil pH (in water) was 7.1 and the electrical conductivity was 1.4 mS cm^−1^. Recommended dose of nitrogen (120 kg N ha^−1^) and potassium (40 kg K_2_O ha^−1^) were added to the soil. Initially, six seeds were sown in each pot (size 30 cm diameter × 30 cm height) which were thinned to four healthy plants after emergence of 3–4 leaves comprising one experimental unit (one pot with four plants). Three such experimental units were used for recording data thus three replicates for each accession and P treatment. The weather data recorded on daily basis for the whole crop duration for two seasons are presented in Supplementary Fig. 1A,B.

**Figure 1:**
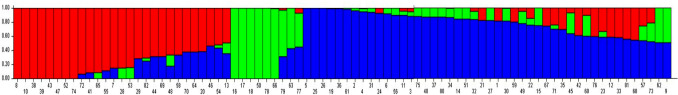
Estimated population structure of 82 wheat accessions (*K* = 3) using STRUCTURE. Three subpopulations were revealed, C1—red color, C2—green color and C3—blue color. The accession number corresponds to the serial number mentioned in Supplementary Table S1.

The accessions were phenotyped for various P efficiency traits. The total biomass (TBM) and grain weight per plant (GWP) were recorded after harvest. Shoot and grain P concentration was estimated after digestion with diacid mixture containing HNO_3_ and HClO_4_ acid in 4:1 ratio following method of Murphy and Riley^40^. The shoot P percentage (SPP), shoot P uptake (SPU), grain P percentage (GPP), grain P uptake (GPU) and total P uptake (TPU) were calculated after analysing P concentration. The P acquisition efficiency (PAE) was calculated as the ratio of P content at low P to sufficient P. P harvest index (PHI) was computed as the ratio of P content in grain to the P content in grain plus shoot. The P use efficiency (PUE) was calculated as the ratio of grain yield to total P uptake^41^. The P stress susceptibility index (PSSI) was calculated according to Eq. 1:

1 $$ {\text{Phosphorus}}\;{\text{stress}}\;{\text{susceptibility}}\;{\text{index}}\;\left( {{\text{PSSI}}} \right) = 1 - \, \left( {{\text{Y}}_{{{\text{LP}}}} /{\text{Y}}_{{{\text{SP}}}} } \right)/1 - \left( {{\text{X}}_{{{\text{LP}}}} /{\text{X}}_{{{\text{SP}}}} } \right) $$

Y_LP_ denotes the mean yield of genotype under low phosphorus (LP) and Y_SP_ the mean yield of genotype under sufficient phosphorus (SP). X_LP_ and X_SP_ denotes the mean Y_LP_ and Y_SP_ respectively of all genotypes.

### Phenotyping in hydroponics

Surface sterilized seeds with 0.1% HgCl_2_ were germinated and after emergence of coleoptiles, seedlings were transferred to full strength nutrient solution at two concentrations of P: 500 µM (sufficient P) and 5 µM (low P). The composition of nutrient solution and method to grow plants were followed as mentioned earlier ^42^. The solution pH was maintained at 5.6 throughout the experiment using 1.0 N HCl or 1.0 N KOH. The growth conditions in the glasshouse were maintained at temperatures 22–12°C/day-night, 10 h photoperiod and 90% relative humidity. Four replications were maintained for each P treatment and accessions. Out of four, two trays were sown at staggered time interval of 15 days which served as repetition over time. Destructive sampling was done after 22 days of growth in nutrient solution and the plants were separated into root and shoot and dried in a hot air oven. Traits such as total biomass (TBM), shoot dry weight (SDW), root dry weight (RDW), root-to-shoot ratio (RSR), P concentration in whole plant (PCON), total P uptake and PAE were recorded.

### Statistical analyses of phenotyping data

The design of experiment was completely randomized (CRD) with two factors, P and accessions. Pooled data was analysed by two-way analysis of variance (ANOVA). Principal component (PCA) and correlation of traits between different subpopulation were carried out using R ver 3.5.1 (R Foundation for Statistical Computing, Vienna) ^43^. Eleven quantitative traits were used to perform PCA and the traits that differentiated accessions for P efficiency were identified by the loading scores. Analysis of genotypic variation and their interaction with traits were carried out in R (GGE Biplot GUI ver 1.0–9). Supplementary Figures S1 and S2 were made in MS Excel. The broad sense heritability (H^2^) for the whole panel of 82 accessions was calculated by the ratio of genotypic variance and phenotypic variance. From ANOVA data, the genotypic variance (GV) and phenotypic variance were calculated.

### Genotyping and properties of SNP markers

The DNA was extracted from 8–10 days old seedlings (10 plants pooled) following CTAB method^44^ and all accessions were genotyped with the 35 K Wheat Breeders Affymetrix-SNP array (Axiom Wheat Breeder’s Genotyping Array;^45^) after standard Axiom sample preparation^46^. SNP allele calling was carried out using the proprietary software package ‘Axiom Analysis Suite’ (Thermo), following the Axiom Best Practices Genotyping Workflow. A total of 35,143 SNPs was scored on the array. Markers were eliminated from the data set if they showed more than 10% missing values or had a minor allele frequency (MAF) of < 5%. After performing these checks, a total of 10,019 high-quality SNP markers remained in the dataset which were used for population structure and diversity analysis. The frequency at which the second most common allele occurs in a given population is MAF while the polymorphic information content (PIC) indicates the genetic properties of SNPs in a population from different aspects and was calculated using the formula given by Botstein et al.^47^.

### Population structure analysis

The population structure was analysed using Bayesian approach. Model-based clustering was performed using software STRUCTURE version 2.3.4^48^. Population structure was evaluated by means of *K*-values (an assumed fixed number of subpopulations) from 1 to 10 in the population. Three independent analyses were used for each *K*-value and the STRUCTURE program was set on 50,000 as burn-in iteration followed by 100,000 Markov Chain Monte Carlo (MCMC) replications after burn-in^49^. The optimal value of *K* was detected by using STRUCTURE HARVESTER v0.6.94^50^ based on ad-hoc method described by Pritchard et al.^48^ in the user manual as well as Evanno’s method^51^. Expected heterozygosity (*He*)^52^, gene diversity (GD)^53^ and fixation index (Fst)^54^ values were derived from STRUCTURE analysis results. Expected heterozygosity is a basic estimate of genetic diversity in a population and describes the expected proportion of heterozygous genotypes under Hardy–Weinberg equilibrium. The Fst is the measure of genetic variance that can be described by population structure based on Wright’s *F*-statistics where a value of 0 indicates no differentiation between the subpopulations while a value of 1 indicates complete differentiation.

### Analysis of molecular variance (AMOVA) and genetic diversity indices

The number of subpopulations determined by STRUCTURE was used for AMOVA and GenAlEx v6.503^55^ to estimate genetic diversity and calculate the diversity indices among the 82 bread wheat accessions. The diversity indices including Shannon’s diversity index (*I*), diversity index (*h*), and unbiased diversity index (*uh*) were calculated for each population. In addition, genetic indices such as number of loci with private allele, number of different alleles (A), number of effective alleles (Ae), percentage of polymorphic loci (PPL), number of private alleles and gene flow or haploid number of migrants (*N*_*m*_) were also calculated using GenAlEx v6.503.

### Genome wide association study for identifying marker-trait association

GWAS was conducted using 31,926 SNPs markers, after removing the missing genotyping calls and very low MAFs, and the best linear unbiased predictors (BLUPs) for phenotypic traits measured under different environments among bread wheat 82 accessions for P efficiency in soil and hydroponics at two P levels. The traits used were TBM, GWP, SPP, SPU, GPP, GPU, TPU, PHI, PSSI and PUE under low and sufficient P soil. Apart from soil experiment, traits like TBM, SDW, RDW, RSR, PCON and TPU were measured under low and sufficient P hydroponics condition. The association mapping was performed by fitting four different models such as general linear model (GLM), mixed linear model (MLM)^56,57^, multiple loci mixed model (MLMM) and Fixed and Random Model Circulating Probability Unification (FarmCPU) methods^58^ to select the best fitting model and reduced false discovery rate (FDR). The FarmCPU uses a fixed effect model (FEM) and a random effect model (REM) iteratively to remove the confounding between testing markers and kinship that results in false negatives, prevents model overfitting, and control false positives simultaneously^58^. Therefore, GWAS was performed on the adjusted BLUPs for each trait in each year to identify SNPs associated with both P levels and efficiency traits using FarmCPU with population structures (Q1 and Q2) or first three principal components (PC1, PC2, and PC3) as covariates. It was done by looking at the model fit using Quantile–Quantile (Q-Q) plots and FarmCPU-calculated kinship implemented in GAPIT (Genome Association and Prediction Integrated Tool) of the R software (with advanced kinship clustering algorithm)^59^. A uniform suggestive genome-wide significance threshold level of *p*-value > (− log10 *p* = 6.00) was selected for MTAs considering the deviation of the observed test statistics values from the expected test statistics values in the Q–Q plots from the two-year results of the present study. Each significant MTA was further subjected to Bonferroni multiple correction (using additional αGWAS ≤ 0.05) for eliminating the false positives.

### Determination of linkage disequilibrium

The GAPIT program in R software was used to conduct linkage disequilibrium analysis^26^. The squared allele frequency correlations (*R*^2^) at *p*-values < 0.001 for each pair of loci were considered to estimate significant linkage disequilibrium. The physical map depicting chromosomal localization of SNP markers was drawn using MapInspect software (https://www.plantbreeding.wur.nl/UK/software_map-inspect.html) (Fig. 7A). The R package CM-plot (ver 3.6.2) was used to create the SNP density plot (Fig. 7B).

### Identification of candidate genes for significant SNPs

In order to identify potential candidate genes, genes within 0.25 Mbp (this distance was chosen on the basis of LD decay value) on both the sides of each MTA were retrieved using BioMart form EnsemblPlant. These were annotated using InterPro and their biological function was analysed using gene ontology available at Biomart of Ensemble plant.

## Results

### SNP distribution on ABD genomes

In the analysis of data, out of total 35,143 SNPs, 0.31% (110) could not be scored as they gave insufficient signal, while 38.5% (13,595) showed more than 10% missing values. Hence, these markers were eliminated from the dataset. Apart from this, 32.5% (11,419) SNPs were excluded because they exhibited a MAF < 5%. After data processing and SNP filtering, a total of 10,019 high-quality SNPs were used for further analysis. The distribution of effective high-quality SNPs was 3271, 3823 and 2925 in the A, B and D genomes, respectively (Supplementary Table S2). Number of effective SNPs per chromosome ranged from 151 (chromosome 4D) to 736 (chromosome 1B). When compared with the total number of high-quality SNPs, percentage of effective SNPs identified were 32.6%, 38.2% and 29.2% in A, B and D genomes respectively.

The PIC values for 10,019 SNP markers varied from 0.090 to 0.375 with a mean of 0.233. The SNP markers in A, B and D genomes exhibited mean PIC values of 0.235, 0.231 and 0.228, respectively (Supplementary Fig. S2A). Among 10,019 SNP markers, 485 (4.85%) had maximum PIC value of 0.375, whereas 83 (0.083%) had minimum PIC value of 0.09 and the remaining 9,451 (94.33%) fell within the range of 0.092 to 0.374. The mean values of PIC across all chromosomes ranged from 0.224 (chromosome 4) to 0.238 (chromosome 2) (Supplementary Fig. S2B).

### Analysis of population structure

The most likely number of structured subpopulations, *K-* value, was used to analyse the number of clusters based on the genotypic data throughout the genome in 82 wheat accessions. Optimal value of *K* and the number of clusters (*K*) was determined by plotting ‘*K*’ against *ΔK* that exhibited a sharp peak at *K* = 3 (Supplementary Fig. S3). A gradual enhancement was observed in the assessed log likelihood[Ln P(D)] with the increase in *K* and optimal number of *K*-value which determined the number of populations as *K* = 3, depicting that three subpopulations could include all 82 accessions with the highest probability. These subpopulations were distinct and consisted of 27, 9 and 46 accessions in C1, C2 and C3 groups, respectively (Fig. 1).

Remarkable genetic divergence was detected among three subpopulations and expected heterozygosity or gene diversity (average distance, *He*) was noted among accessions in each subpopulation. Highest value of *He* was observed in C2 and the lowest was noted in C1 (Table 1). The STRUCTURE results estimated Fst for each subpopulation indicating significant divergence within these subpopulations. The Fst value was highest in C1 and lowest in C2 while C3 exhibited moderate Fst value (Table 1).

**Table 1:** The STRUCTURE analysis of 82 wheat accessions for the fixation index (Fst), expected heterozygosity (*He*) and number of accessions assigned to each subpopulation.

Subpopulations	Fst	*He*	No. of accessions
C1	0.538	0.250	27
C2	0.344	0.364	9
C3	0.403	0.267	46

### Analysis of genetic variations in subpopulations

Within and among three subpopulations, the components of total genetic variation were calculated by AMOVA (Table 2). The AMOVA showed 14% of total variation among subpopulations, whereas the rest of the variation (86%) was within the subpopulations, suggesting a high level of differentiation. The Nm (haploid number of migrants) value of 3.106 indicated a higher genetic exchange or gene flow among subpopulations resulting in poor population differentiation. These results confirmed that the genetic variation among subpopulations was low as compared to the genetic variation within subpopulations.

**Table 2:** Analysis of molecular variance (AMOVA) using 10,019 SNPs of the genetic variation among and within the subpopulations of 82 wheat accessions.

Source	df	SS	MS	Est. Var	%
Among Pops	2	17,736.107	8868.053	301.935	14%
Within Pops	79	148,179.113	1875.685	1875.685	86%
Total	81	165,915.220		2177.620	100%
Nm (Haploid)	3.106				

Through STRUTURE analysis, it was observed that the accession pedigree is the main factor for the groupings. It was noted that most of the accessions from India belonged to C3, while those from Mexico and Australia were grouped under C1 subpopulation. The Mexican accessions grouped under C1 included ‘ATTILA’, ‘BABAX’, ‘CULIACANT 89′ and ‘VEE/MYNA’. The Australian accessions such as ‘BENCUBBIN’, ‘BLADE’, ‘BROOKTON’, ‘CALINGIRI’, ‘CARNAMAH’, ‘DATATINE’, ‘EGRET’, ‘STILLETO’, ‘TAMMIN’ and ‘WARIGAL’ were also grouped under C1 subpopulation. Subpopulation C3 contained most of the advanced lines from CIMMYT and crosses made in the country consisting of CIMMYT parents. Accessions grouped under subpopulation C2 includes ‘EC 576,621’, ‘GUTHA’, ‘IC 534,271’, ‘TURACO’, ‘STRETTON’, ‘OLYMPIC’, ‘SAMNGP 402’, ‘SAMNGP 404’ and ‘SAMNGP 407’. Among subpopulations, no landrace was present in C1 while in C2, there were three (SAMNGP 402, SAMNGP 404 and SAMNGP 407) landraces.

### Genetic diversity and allelic pattern across the populations

The intra-population genetic diversity analysis revealed the A and Ae allele numbers as 2.195 and 1.570, respectively (Supplementary Table S3). The highest number of observed alleles (A = 2.512) were found in C3, while the lowest number was recorded in C2 (A = 1.981). Moreover, the maximum number of effective alleles were found in the C2 subpopulation (Ae = 1.665).

The average values of genetic diversity indices such as Shannon’s information index (*I*), diversity index (*h*), and unbiased diversity index (uh) were 0.503, 0.313, and 0.335, respectively. The Shannon diversity index value varies between 0 and 1, where 1 indicates complete evenness. We found *I* values of different subpopulations ranged from 0.453 (C1) to 0.530 (C2) whereas *h* ranged from 0.280 (C1) to 0.349 (C2). Among the three subpopulations, C2 was highly diverse (I = 0.530, h = 0.349, uh = 0.395), C1 was least diverse (I = 0.453, h = 0.280, uh = 0.292) while C3 exhibited moderate values of diversity indices (I = 0.526, h = 0.310, uh = 0.318). Further, C3 subpopulation exhibited the highest (96.73%) percentage of polymorphic loci (PPL) while lowest (82.30%) was noted in C2. The lowest private allele number was observed in C1 whereas C2 and C3 showed higher values of number private alleles. Thus, SNPs can act as useful markers for genetic diversity studies.

### Principal component analysis of phenotypic traits of different subpopulations

**Figure 2:**
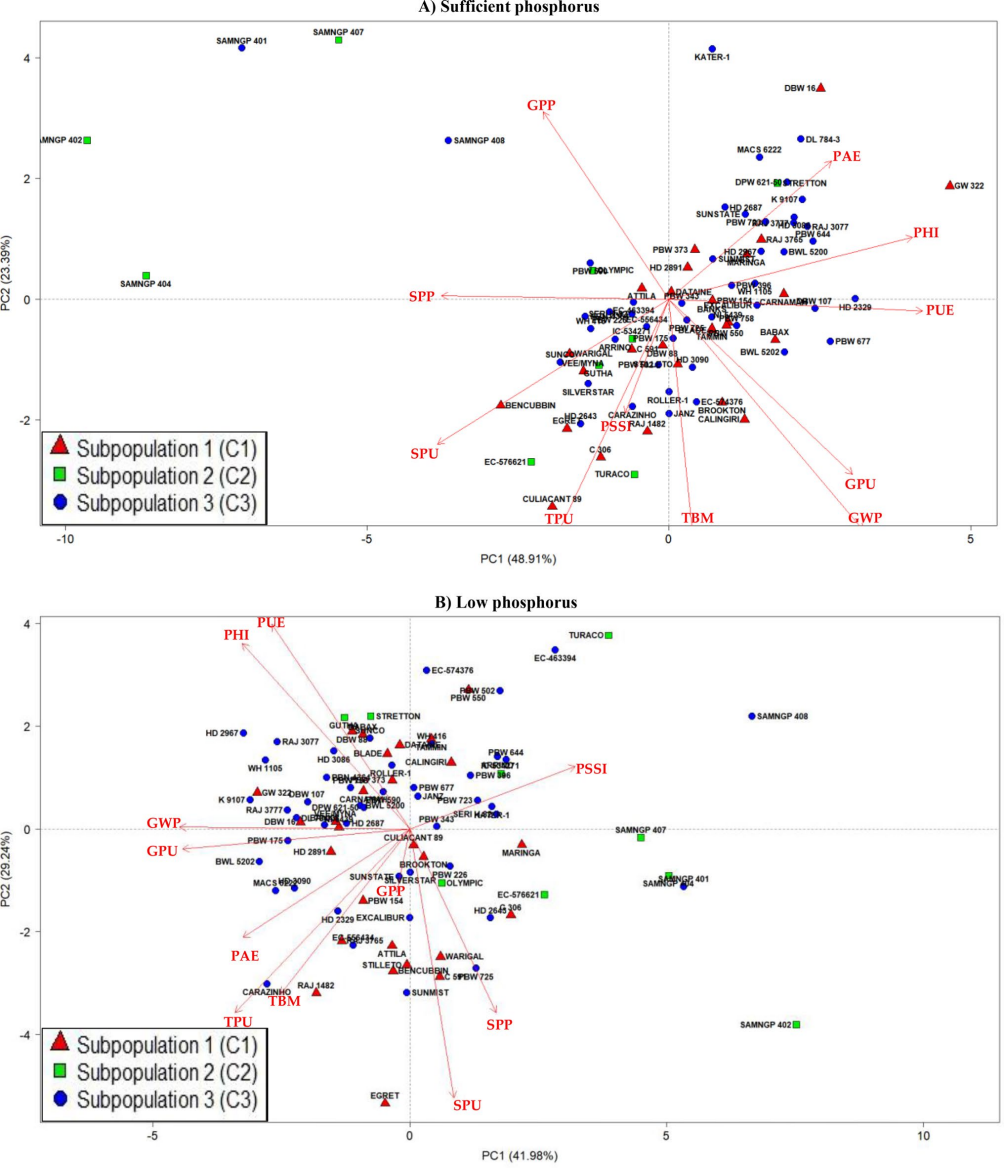
Biplots at (**A**) sufficient (42.2 mg P kg^−1^ soil), and (**B**) low (2.67 mg P kg^−1^ soil) phosphorus. The subpopulations C1, C2 and C3 were derived based on the SNP markers. Abbreviations: TBM, total biomass per plant; GWP, grain weight per plant; SPP, shoot P percentage; SPU, Shoot P uptake; GPP, grain P percentage; GPU, grain P uptake; TPU, total P uptake; PHI, P harvest index; PAE, P acquisition efficiency; PUE, P use efficiency; PSSI, P stress susceptibility index.

The pooled mean (two years) for all traits recorded on 82 wheat accessions were subjected to PCA at sufficient and low P (Fig. 2A,B). The biplot analysis showed that PC1 and PC2 governed 72.3% variability respectively at sufficient P (Fig. 2A) while PC1 and PC2 governed 71.2% of variability at low P (Fig. 2B).

Traits which explained genotypic variability at sufficient P in PC1 were PUE (17.6%), PHI (16.3%), shoot P uptake (14.7%) and P percentage in shoot (14.2%) (Supplementary Table S4). Under low P, traits such as grain weight per plant (20.1%) and grain P uptake (19.6%) were strongly associated with population structure. Within PC2, the strong association with population structure was shown by total biomass (28.9%), total P uptake (22.6%) and grain weight per plant (14.7%) at sufficient P. Similarly, in PC2, the genotypic variation was governed by shoot P uptake (27.4%) and PUE (15.9%) at low P.

### Growth response of subpopulations to phosphorus levels in soil and hydroponics

Traits were recorded for two consecutive years for soil experiment and pooled data was subjected to analysis of variance. Significant (*P* < 0.001) effect of genotype and P levels were observed for various P efficiency traits in wheat accessions (Supplementary Tables S5). The reduction in total biomass and grain weight per plant at low P was 20 and 24%, respectively. Total P uptake decreased significantly by 49% at low P in comparison to sufficient P. However, a significant increase was noted in PHI (18%) and PUE (45%) at low P. The *H*^*2*^ ranged from 83.4% (total P uptake) to 94.9% (total biomass). The average values subpopulation-wise for various phenotypic traits recorded for years 2016 and 2017 showed significant (P < 0.001) effect in all subpopulations except for PHI in 2016 (Supplementary Tables S6 and S7). Further, the pooled data over two years showed significant (P < 0.001) effect in all subpopulations (Table 3). The genetic variance and broad sense heritability (*H*^*2*^) were estimated for each trait for all subpopulations. The genetic variance for most of the traits was found to be highest in C2. However, C3 subpopulation showed the minimum values of genetic variance for most of the traits while C1 was intermediate. The *H*^*2*^ ranged from 70% (grain weight per plant) to 92.9% (PHI) in C1, from 86% (total P uptake) to 98.37% (grain weight per plant) in C2, and from 81.8% (grain P percentage) to 93.79% (grain weight per plant) in C3 subpopulation. For most of the traits, the *H*^*2*^ was highest in the genetically diverse C2 subpopulations as compared to the other two subgroups. The descriptive statistics shows the mean, range, standard deviation and coefficient of variation (CV) for each trait under low and sufficient P levels in C1, C2, and C3 subpopulations (Supplementary Table S8 A, B and C). The CV values in C1 for both P levels were generally low, except for trait like shoot P uptake where it was > 20%. Similarly, in C3, the CV values were < 20% except for shoot P concentration and shoot P uptake. However, the CV values in C2 showed more diversity ranging from 14.28 to 34.57% under sufficient P, and from 9.67 to 33.03% under low P treatment. The relative values (ratio of mean values at low P with mean values at sufficient P) of most of the physiological traits were higher in C3 subpopulation followed by C2 while lowest was observed in C1. Also, the minimum and maximum range for these traits at sufficient and low P were higher in C3 followed by C2 subpopulation.

**Table 3:** ANOVA, genetic variance (GV) and broad sense heritability (*H*^*2*^) for P use efficiency related traits recorded in three subpopulations (C1, C2 and C3), obtained from 82 bread wheat accessions, grown under sufficient (42.2 mg P kg^−1^ soil) and low (2.67 mg P kg^−1^ soil) phosphorus soil. The pooled data over two years (2016 and 2017) was used for analysis. Abbreviations: TBM, total biomass plant^−1^; GWP, grain weight plant^−1^; SPP, shoot P percentage; SPU, shoot P uptake; GPP, grain P percentage; GPU, grain P uptake; TPU, total P uptake; PHI, P harvest index; PUE, P use efficiency. * *P* < .05; ** *P* < .01, *** *P* < .001; n.s., non-significant; P – phosphorus; G – accession; PXG – interaction between P and G.

Traits	C1 Subpopulation					C2 Subpopulation					C3 Subpopulation				
	*F*- value			GV	*H*^*2*^(%)	*F*- value			GV	*H*^*2*^(%)	*F*- value			GV	*H*^*2*^(%)
	P	G	PXG			P	G	PXG			P	G	PXG		
TBM	853.54***	35.42 ***	8.61***	15.43	92.23	586.20***	69.81***	12.22***	25.87	95.92	1707.82***	44.76***	8.36***	16.20	93.64
GWP	964.43 ***	7.72***	5.79***	0.93	70.00	453.37***	182.87***	25.37***	14.51	98.37	1663.03***	40.32***	6.17***	4.53	93.79
SPP	2467.81***	12.10***	7.67***	0.00	75.00	1634.06***	76.70***	35.42***	0.01	95.73	5498.65***	22.77***	18.74***	0.00	88.24
SPU	2282.19***	29.21***	9.99***	86.33	90.56	1237.34***	40.78***	14.34***	179.00	92.75	5169.07***	23.98***	13.98***	47.00	88.68
GPP	783.97***	12.85***	8.86***	0.00	79.44	663.41***	54.30***	15.89***	0.00	94.66	2076.22***	17.46***	5.44***	0.00	81.82
GPU	1971.36***	10.10***	4.63***	19.67	76.62	1026.19***	83.36***	20.77***	105.33	96.34	3348.31***	25.53***	4.43***	53.00	88.33
TPU	3812.12***	14.46***	5.56***	78.00	82.11	2542.90***	18.75***	19.12***	86.00	86.00	8203.73***	17.01***	8.37***	69.00	84.15
PHI	532.56***	38.37***	8.05***	92.00	92.93	170.27***	67.95***	2.81*	305.67	95.71	822.65***	28.04***	7.27***	76.00	90.48
PUE	1692.44***	26.05***	7.73***	7.97	88.85	574.20***	84.59***	5.04***	43.07	96.63	4214.10***	28.70***	8.73***	7.33	88.00

In hydroponics screening, the genotype and P levels significantly (*P* < 0.001) influenced the various traits in wheat accessions (Supplementary Tables S9). Total biomass, shoot dry weight and total P uptake decreased significantly but root dry weight and root-to-shoot ratio increased at low P compared to sufficient P concentration. Total P uptake was reduced by more than 72% at low P concentration in wheat and triticale genotypes in comparison to sufficient P. The *H*^*2*^ ranged from 72.15% (root-to-shoot ratio) to 100% (P concentration). The subpopulations C1, C2, and C3 showed significant (*P* < 0.001) effect of P and accessions for all traits (Table 4). The genetic variance was higher in C2 for almost all traits except for root dry weight as compared to other two subgroups. The *H*^*2*^ varied from 77.59% (root-to-shoot-ratio) to 97.96% (total biomass) in C2, from 78.98% (root-to-shoot-ratio) to 100% (P concentration) in C1, and from 75.61% (root-to-shoot-ratio) to 100% (P concentration) in C3. Similarly, the highest *H*^*2*^ was recorded in the genetically diverse C2 subpopulation for most of the traits. The descriptive statistics showed that the CV values obtained were very high (> 30%) in all subpopulations and not acceptable (Supplementary Table S10A, B and C). In C3, the CV values were < 30% and were acceptable except for root dry weight under sufficient P. However, root-to-shoot ratio and P concentration were < 20% at sufficient P and < 30% at low P in C1 and C2, respectively. Among subpopulations, C2 showed maximum increase in root dry weight (42%) and root-to-shoot ratio (114%) at low P as compared to sufficient P.

**Table 4:** ANOVA, genetic variance (GV) and broad sense heritability (*H*^*2*^) for P use efficiency related traits recorded in three subpopulations (C1, C2 and C3), obtained from 82 bread wheat accessions, grown under sufficient (500 µM) and low (5 µM) concentration in hydroponics. Abbreviations: TBM, total biomass; SDW, shoot dry weight; RDW, root dry weight; RSR, root-to-shoot ratio; PCON, phosphorus concentration; TPU, total phosphorus uptake. *P < .05; ** *P* < .01, *** *P* < .001; P – phosphorus; G – accession; PXG – interaction between P and G.

Traits	C1 Subpopulation					C2 Subpopulation					C3 Subpopulation				
	*F*- value			GV	*H*^*2*^(%)	*F*- value			GV	*H*^*2*^(%)	*F*- value			GV	*H*^*2*^(%)
	P	G	PXG			P	G	PXG			P	G	PXG		
TBM	153.63***	76.67***	5.02***	0.02	96.18	132.65***	144.02***	8.93***	0.02	97.96	210.16***	52.63***	5.31***	0.01	94.48
SDW	230.11***	56.62***	5.05***	0.01	94.86	197.28**	116.55***	9.26***	0.02	97.48	363.84***	39.94***	5.09***	0.01	92.84
RDW	225.35***	114.22***	9.08***	0.00	97.35	159.80***	71.10***	6.34***	0.00	95.90	700.50***	65.01***	12.86***	0.00	95.64
RSR	404.81***	11.91***	11.82***	0.00	78.98	232.33***	11.68***	5.75***	0.01	77.59	947.36***	10.55***	8.95***	0.00	75.61
PCON	22,044.92***	31.11***	22.89***	0.00	100.00	7878.24 ***	29.66***	28.19***	0.00	89.87	30,303.84***	10.87***	14.41***	0.00	100.00
TPU	4396.85***	37.48***	20.82***	0.13	92.73	1857.38 ***	95.30***	66.04***	0.21	95.85	7266.66***	35.46***	17.22***	0.09	90.00

The performance of individual accessions in different subpopulations were assessed based on the traits such as total grain weight, shoot P uptake, total P uptake, PUE and PHI obtained from PCA in low P soil contributing > 20% to the genetic variability (Supplementary Table S4). In C1, the genetically least diverse subpopulation, EGRET and ‘NI 5439’ exhibited values higher than the average of subpopulation for these traits (Supplementary Table S8A). In the most genetically diverse subpopulation C2, it was found that out of nine accessions, EC 576,621, GUTHA, IC 534,271, TURACO, and OLYMPIC exhibited values higher than the subpopulation average (Supplementary Table S8B). Similarly, in the moderately diverse C3 subpopulation, the accessions ‘CARAZINHO’, ‘DL 784–3’, ‘PBW 175’, ‘HD 2967’, and ‘RAJ 3777’ possessed values higher than the subpopulation average at low P (Supplementary Table S8C). On the other hand, in hydroponics screen, BABAX, CARNAMAH, ‘DBW 16’, ‘DBW 88’, ‘GW 322’ and NI 5439 belonging to subpopulation C1 showed higher values than the subpopulation mean for total biomass, shoot dry weight, root dry weight, root-to-shoot ratio and total P uptake (Supplementary Table S10A). In the subpopulation C2, accessions IC 534,271, STRETTON, and TURACO exhibited higher values (Supplementary Table S10B) whereas in C3, the accessions DBW 107, CARAZINHO, DL 784–3, EC 574,376, HD 2643, ‘PBW 644’ and RAJ 3777 performed better than the subpopulation average (Supplementary Table S10C). Thus, a few common accessions were identified from the soil and hydroponics experiments in each subpopulation which performed relatively better under low P condition.

### Correlation of phenotypic traits among different subpopulations

**Figure 3:**
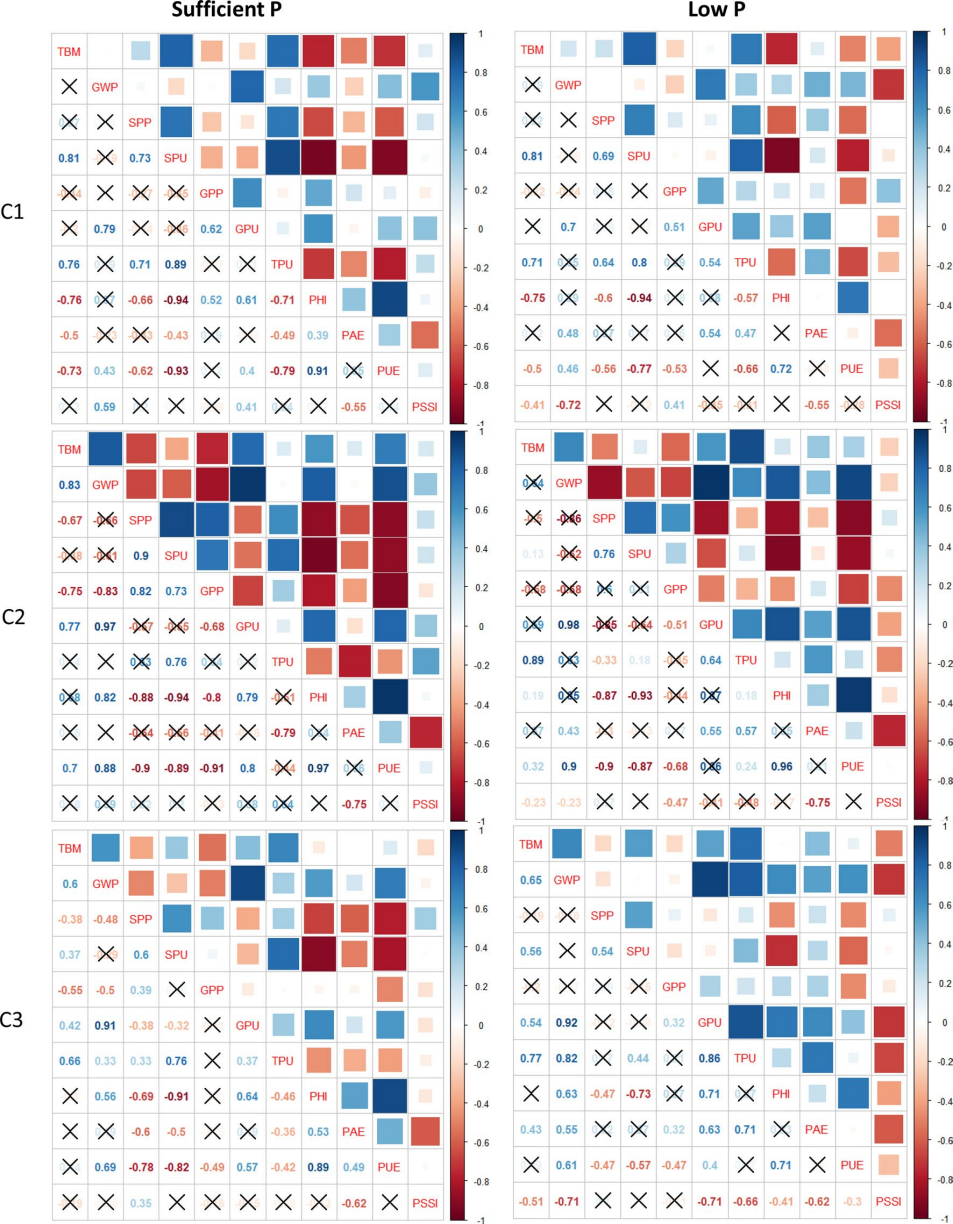
Correlation matrix showing association of three subpopulations, C1, C2, and C3, obtained using SNP markers in 82 diverse wheat accessions and phenotyped in soil under sufficient P and low P. Correlations with p-value > 0.01 are considered as insignificant, such correlation coefficient values are represented with a cross. Abbreviations: TBM, total biomass per plant; GWP, grain weight per plant; SPP, shoot P percentage; SPU, Shoot P uptake; GPP, grain P percentage; GPU, grain P uptake; TPU, total P uptake; PHI, P harvest index; PAE, P acquisition efficiency; PUE, P use efficiency; PSSI, P stress susceptibility index.

**Figure 4:**
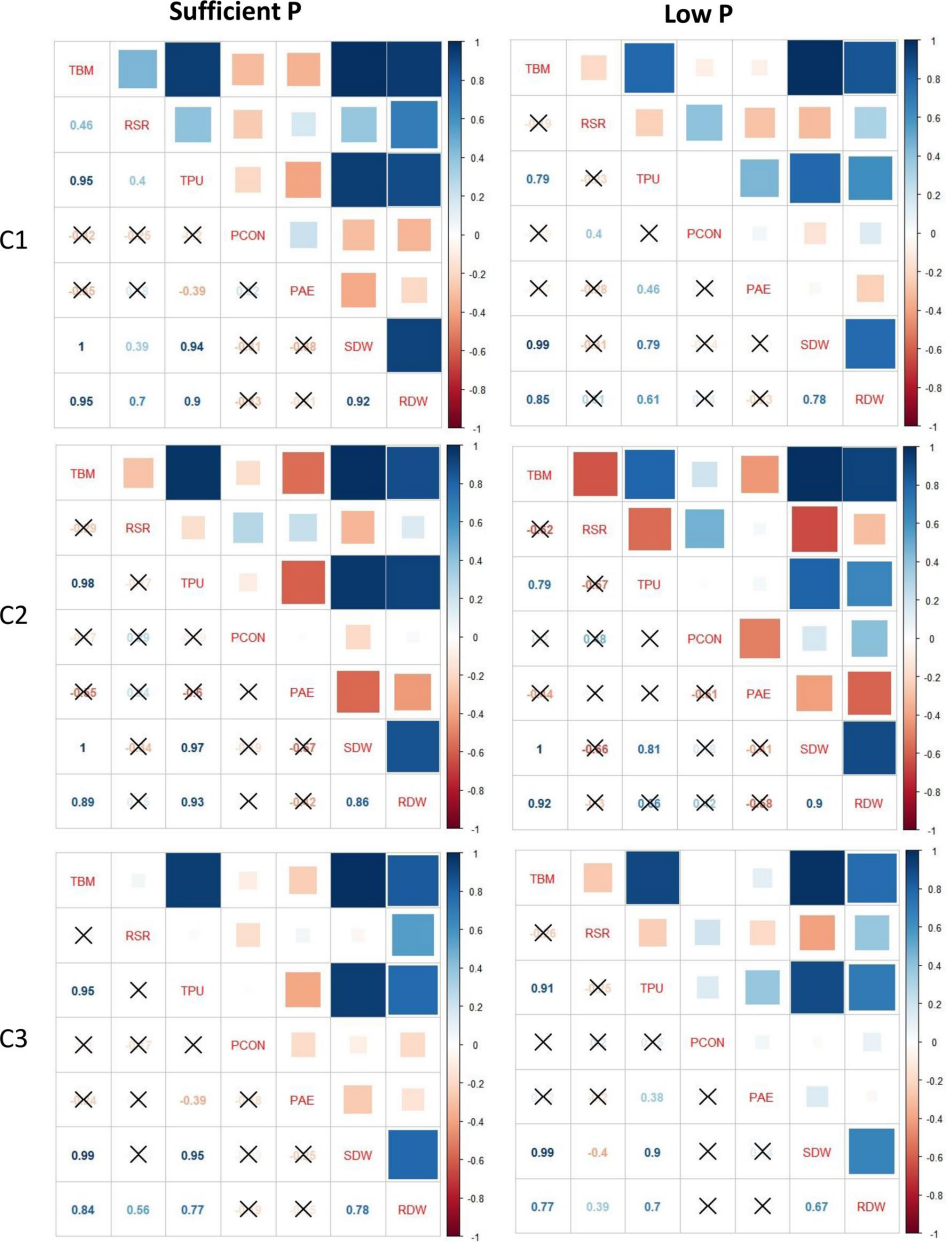
Correlation matrix showing association of three subpopulations, C1, C2, and C3, obtained using SNP markers in 82 diverse wheat accessions and phenotyped under sufficient P and low P concentration in hydroponics. Correlations with p-value > 0.01 are considered as insignificant, such correlation coefficient values are represented with a cross. Abbreviations: TBM, total biomass; RSR, root-to-shoot ratio; TPU, total phosphorus uptake; PCON, phosphorus concentration; PAE, P acquisition efficiency; SDW, shoot dry weight; RDW, root dry weight.

The Pearson’s correlation coefficient revealed the association between various growth and yield traits amongst the subpopulations under sufficient and low P. Compared with subpopulations C1 and C3, the traits in C2 exhibited highly significant (P < 0.05) positive or negative association in both soil (Fig. 3) and hydroponics (Fig. 4) experiments. In soil experiments in the C2 subpopulation, the grain weight per plant showed higher correlation with PUE (r = 0.88) and total biomass (r = 0.83), the shoot P concentration exhibited correlation with shoot P uptake (r = 0.90) and grain P concentration (r = 0.82) and between PHI and PUE (r = 0.97) under sufficient P. In low P soil, C2 subpopulation showed higher correlation for grain weight per plant with PUE (r = 0.90) and grain P uptake (r = 0.98), and between PHI and PUE (r = 0.96) (Fig. 3). Likewise, in the hydroponically grown plants, the correlation values were higher in C2 subpopulation as compared to C1 and C3 under both sufficient and low P condition (Fig. 4).

### Marker-trait association

**Figure 5:**
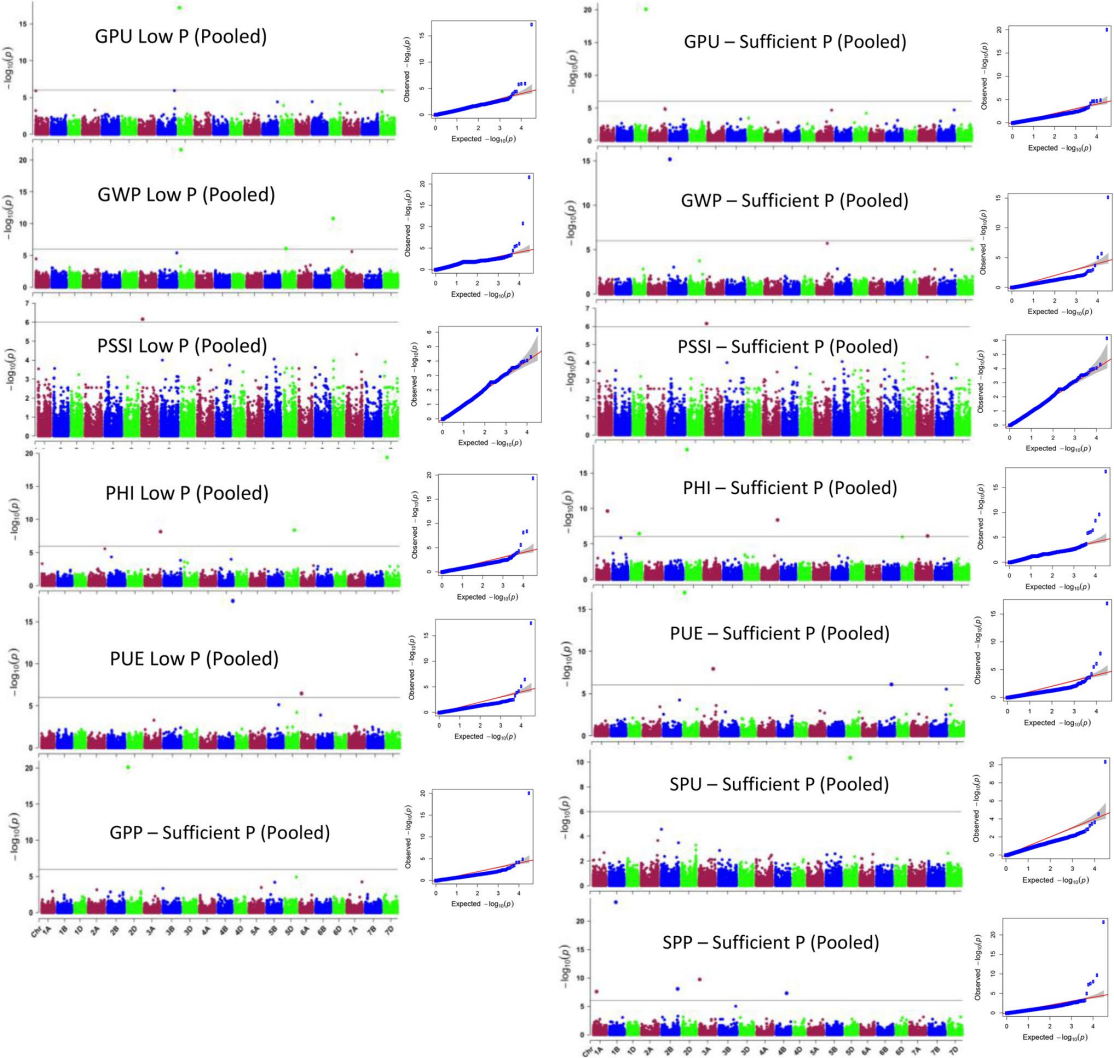
Manhattan plots showing SNP markers associated with different traits and Quantile–Quantile plots for 82 bread wheat accessions grown in low P soil using data pooled for two years. The horizontal line represents FDR adjusted p < 0.001. The threshold of -log_10_ (*P*-value) ≥ 6.0 was used as a cutoff to identify association analysis. Abbreviations: GPU, grain P uptake; GWP, grain weight per plant; PSSI, P stress susceptibility index; PHI, P harvest index; PUE, P use efficiency; GPP, grain P percentage; SPU, Shoot P uptake; SPP, shoot P percentage.

**Figure 6:**
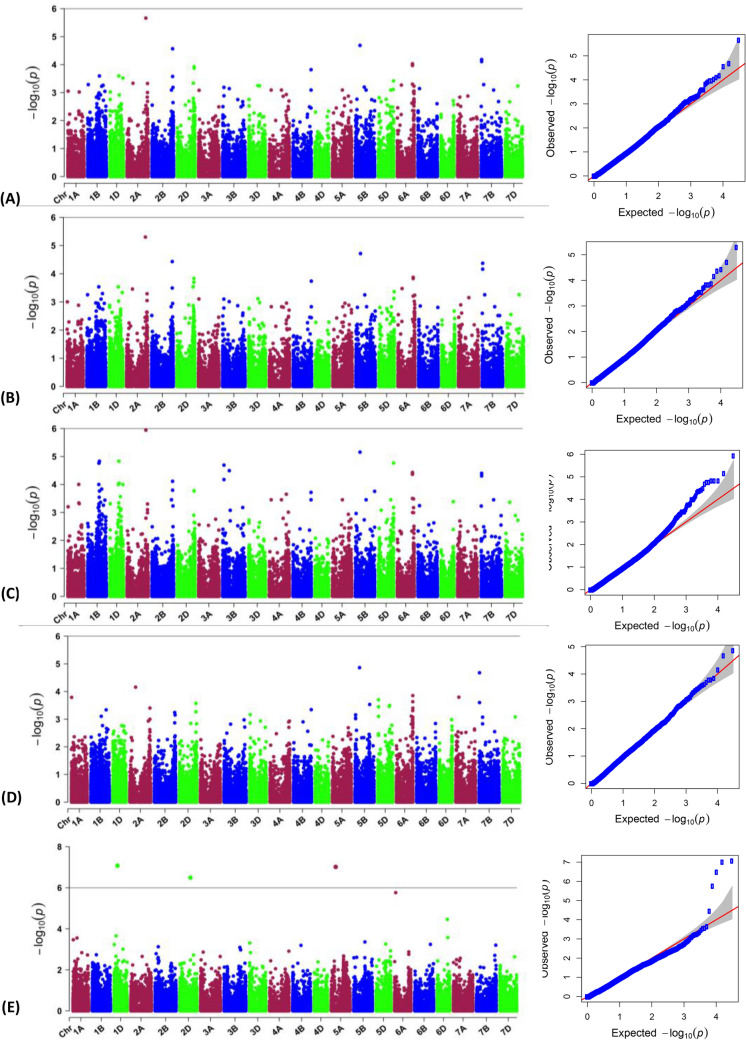
Manhattan plots showing SNP markers associated with different traits and Quantile–Quantile plots for 82 bread wheat accessions grown in hydroponics. **(A**) Shoot dry weight, (**B**) Total biomass, and (**C**) Total P uptake, at sufficient phosphorus level, and (**D**) Total biomass, (**E**) P concentration, at low P level. The horizontal green line represents FDR adjusted p < 0.001. The threshold of −log_10_ (*P*-value) ≥ 6.0 was used as a cutoff to identify association analysis.

**Figure 7:**
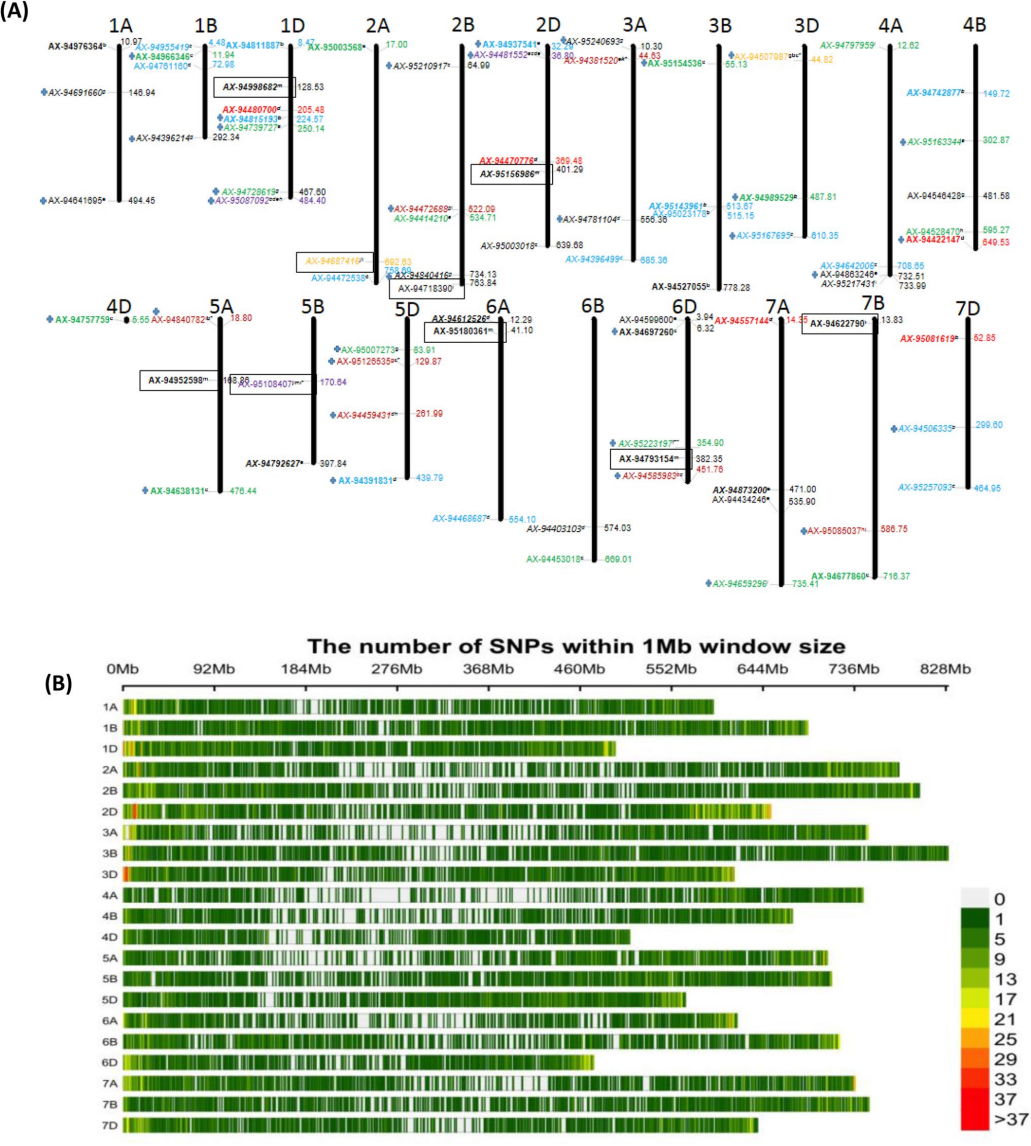
**(A)** Chromosomal localization of SNP markers on 21 chromosomes of wheat. The chromosome numbers are indicated on top of chromosomes. On each chromosome, the marker names are indicated on the right side and their physical positions are indicated on the left side. Markers without a box: identified under soil; markers within a box: identified under hydroponics; a-m: different traits (a-GPP, b-GPU, c-GWP, d-PUE, e-PHI, f-PAE, g-SPP, h-SPU, i-TBM, j-TPU, k-PSSI, l-SDW and m-PCON); normal font: markers under sufficient P; Bold font: markers under low P; Italic font: markers for which genes identified, markers with *: available in sufficient P and low P. Different colours indicate markers in different situations and for different traits: Blue: 2016; Green: 2017, Red: 2016 and pooled data (2016 + 2017); Purple, Orange, Maroon and Black: markers identified for more than four, three, two and one trait. Markers with blue plus sign passed Bonferroni Correction. **(B)** SNP density plot chromosome wise representing number of SNPs within 1 Mb window size. The horizontal axis shows the chromosome length (Mb); the different color depicts SNP density.

A total of 78 MTAs (having *P*-value less than 0.0001) were found in both, soil (Fig. 5) and hydroponics (Fig. 6) experiments under sufficient P (SP) and low P (LP) levels for 13 different quantitative traits (11 traits in soil and four traits in hydroponics; two were common) (Supplementary Fig. S4–S5). Out of 78 SNPs, 69 unique MTAs were identified in soil experiment (47 MTAs under SP and 27 MTAs under LP; five were common between SP and LP though the traits were different for three MTAs) (Supplementary Table S11) whereas 9 unique MTAs were identified in hydroponics experiment (3 MTAs under SP and 7 MTAs under LP; one was common between SP and LP for TBM) (Supplementary Table S12). Maximum number of significant MTAs having *P*-value less than 0.0001 were present on D (28) genome followed by B (26) and A (24) genome (Fig. 7A).

The significant MTAs identified in soil experiment were associated with all 11 traits such as TBM (3), GWP (9), SPP (10), SPU (8), GPP (4), GPU (4), TPU (3), PHI (8), PUE (9), PSSI (1), PAE (1) under SP while with six of eleven traits, that is, GPU (10), GWP (8), PAE (1), PUE (5), PHI (4) and PSSI (1) under LP. Similarly, in hydroponics experiment, the significant MTAs were associated with three of seven traits, they were TPU (1), SDW (2) and TBM (3) in SP and two of seven traits were PCON (5) and TBM (2) in LP. No significant MTAs were found for RDW and RSR in hydroponics culture. Out of 78 MTAs, only 35 MTAs passed Bonferroni correction which belonged to soil experiment only (Fig. 7A) and were used for further analysis. From these 35 MTAs, we found two such MTAs which were regulating four traits (AX-95087092 on chromosome 1B: GPU, PUE, PHI, SPU; AX-94481552 on chromosome 2D: GPP, GWP, PUE, PHI), one MTA controlling three traits (AX-94507987: SPP, GPU. GWP) and six MTAs controlling two traits (AX-94472688 on 2B: SPP, TBM; AX-94840782: GPU, TBM; AX-95126535: SPP, GWP; AX-94459431 on 5D: PUE, SPU; AX-94585983 on 6D: GPU, GWP and AX-95085037: SPU, TBM) (Fig. 7A).

A total of 297 unique genes within 0.50 Mbp region (0.25 Mbp on both the sides of each MTA) of the 35 MTAs were identified. Out of 297, protein functions were available for 254 candidate genes which mainly belonged to carbohydrate metabolic process, fatty acid biosynthesis, protein phosphorylation/ dephosphorylation, vascular/transmembrane/protein transport and nucleotide biosynthetic process (Supplementary Table S13). The SNP tag sequence of six of nine multi-trait MTAs had 100% identity with high confidence wheat genes (included in 297 genes) which encode proteins for CTP synthase, importin-beta, FAD/NAD(P)-binding domain, Tetratricopeptide-like helical domain, protein binding domain and Ser/Thr kinase which were involved in CTP synthesis, protein transport, oxidation–reduction, hydrolase activity, protein binding and signal transduction, respectively (Supplementary Table 13).

## Discussion

### Genome properties

In this study, we used a 35 K SNP array to genotype and analyse 82 bread wheat accessions. The distribution of effective high-quality SNP markers in the A, B and D genomes scored in the present study were in agreement with earlier reports^1,60^. The maximum number of SNPs were mapped to B genome followed by A and D genomes ^61^. Chromosome 1B had the highest number of high-quality SNPs while chromosome 4D had the lowest number of high-quality SNPs. Greater sequence diversity in A and B genomes than in D genome is the result of gene flow which could have taken place between *Triticum aestivum* and its tetraploid progenitor *Triticum turgidum* (AABB) but are less likely to have been between *Triticum aestivum* and *Aegilops tauschii* (DD)^62^. Earlier reports also provide evidence of lower degree of polymorphism in D genome^60,61,63^. The *T. turgidum* genome likely existed for 200,000 years prior to the addition of the D genome to become *T. aestivum,* this allowed it more time to develop diversity.

The PIC values are very effective for genetic studies in wheat breeding programs as it assesses the genetic variability among accessions. The PIC values are a sign of informative markers within a set of samples which can be utilized for analysing genetic diversity^64^. Botstein et al.^47^ classified markers on the basis of PIC values into three categories-highly informative (PIC > 0.5), moderately informative (0.25 < PIC < 0.5), and slightly informative (PIC < 0.25). The PIC value of SNP marker was found to be moderate or low informative due to bi-allelic nature of SNPs confined to maximum PIC values of 0.50. In our study, the PIC value ranged from 0.090 to 0.375, with an average PIC value of 0.233 based on the selected high-quality effective SNPs, which is in agreement with the PIC value observed for a set of US wheat genotypes^61^. Würschum et al.^60^ reported 1,395 high quality SNP markers with average PIC value of 0.330 in winter wheat. Similar PIC value of 0.240 was observed for wheat population genotyped using 9 K SNP array^65^. Further, in F_3:6_ Nebraska winter wheat populations genotyped with 25,566 SNPs by GBS, an average PIC value of 0.250 was reported^66^.

### Genetic variation among and within the populations assessed with SNP markers

Information regarding genetic diversity and population structure is important for characterizing the domestication history and genetic relatedness of wheat accessions. In our study, analysis of population structure resulted in three subpopulations. Population structure analysis indicated that wheat accessions can be categorized by geographical origin or be divided into landraces and modern varieties. The Fst value obtained for differentiation among three subpopulations was within the acceptable limit which agrees with earlier report stating that Fst value greater than 0.150 can be considered as significant in differentiating populations^67^. Further, lowest Fst value observed in C2, and highest Fst value in C1 indicates that accessions in the latter subpopulation have several different genotypic patterns or due to hybridisation with divergent parent as it is exhibited in allelic richness. Thus, the wheat accessions showed significant divergence within each of the subpopulations.

The gene diversity (*He*) value depends on the allele richness and abundance of alleles in a population. It provides an estimate of the average heterozygosity and genetic distance among the genotypes in a population^68^. The C2 subpopulation exhibited highest *He* value indicating that it possessed greatest genetic diversity within the cluster as compared to C3 and C1. Further, the diversity index and Shannon’s diversity index were lowest in C1 and greatest in C2. Moreover, the lowest private allele number was observed in the C1 whereas C2 containing landraces showed a higher value of private alleles. Among subpopulations, in C1 no landrace was present while in C2, there were three (SAMNGP 402, SAMNGP 404 and SAMNGP 407) landraces thus exhibiting highest level of genetic diversity. This result is in accordance with previous reports in Iranian wheat where the landraces group showed high level of genetic diversity compared with the cultivar group^63^. The adaptability of landraces could be associated with this genetic diversity^69^. Lower level of genetic diversity is correlated with lower number of landraces as reported in a study with 90 Chinese winter wheat accessions that included 11 landraces^70^. Loss of biodiversity has occurred mainly due to substitution of traditional landraces in farmer’s fields with improved cultivars that has limited the wheat genetic diversity. Wild species plays a major role in genetic improvement of cultivars and conservation of genetic resources for crop breeding. Wheat landraces are important sources to widen the genetic base of cultivated wheat. Therefore, landrace populations can be effectively utilized for development of new cultivars to obtain adaptation to climate extremes and tolerance to abiotic and biotic stresses^71^. Further, inclusion of landraces in breeding program could enhance the genetic diversity for quality traits^72^.

The AMOVA and genetic diversity indices calculated for subpopulations showed that within the population, diversity explained most of the genetic diversity as compared to among population diversity. Low level of genetic diversity among subpopulations was related to higher level of gene flow^73^. According to Wright^74^, Nm (haploid number of migrants) value < 1.0 is associated with limited genetic exchange among subpopulations that finally leads to high level of genetic differentiation. In the present study, Nm was high (3.106) which led to gene exchange among subpopulations thereby resulting in low level of genetic differentiation among subpopulations. This result is in agreement with others^66^ who also reported that high Nm value is associated with high gene flow in wheat. Heritability of traits such as plant height, spike number per plant and thousand grain weight reported in wheat crop in field experiment for P efficiency is in close agreement with our results^75^. Similarly, in hydroponics, the heritability of traits such as shoot dry weight per plant, root dry weight per plant and total dry weight per plant agrees with our results.

### Genetic and phenotypic association in wheat population

We further assessed the phenotypic performance of subpopulations, obtained by genetic analysis, for P use efficiency traits. Analysis of phenotypic data derived from soil and hydroponic experiments showed that the highest value of genetic variance and *H*^*2*^ was observed in C2 subpopulations for various traits as compared to C1 and C3. Further, the C2 subpopulation showed very strong correlation values between the traits under both sufficient P and low P in soil as well as hydroponics medium. Nevertheless, we found a few superior genotypes which performed better under low P stress and were common between soil and hydroponic screening in each subpopulation. For example, in C1 subpopulation, NI 5439 was a good performer; in C2, IC 534,271 and TURACO; and in C3 subpopulation, CARAZINHO, DL 784–3, and RAJ 3777 were superior accessions. In earlier reports, CARAZINHO was shown to possess the highest P utilization efficiency for dry matter at low P^76,77^. The genotypes identified in C2 (highly diverse) and C3 (moderately diverse) subpopulations, thus, may be included in the breeding programs to develop new cultivars tolerant to low P stress.

### Marker-trait association

In the present study, we found nine MTAs (having significant *P*-value and passed Bonferroni correction) controlling two to four traits simultaneously. The high confidence candidate genes were identified for six of these nine MTAs. Some of these genes are known to be affected under low P conditions. For instance, FAD/NAD(P) are involved in plant photosynthesis, protein kinases participate in signal transduction and are known to be differentially expressed under P deficiency (Supplementary Table S13)^78,79^. MTAs each controlling two or more than two correlated traits could be recommended for marker-assisted selection (MAS). These MTAs will help not only in the study of genetic architecture of correlated traits, but also in simultaneous improvement of more than two correlated traits through MAS. Though it is not easy to find out whether such multi-trait MTAs are due to pleiotropism or due to close linkage^49^. The stability of an MTA across different years is very important. In the present study, the MTAs which were associated with a particular trait were different in different years. For instance, we found eleven unique MTAs in 2016 and 19 unique MTAs in 2017 under SP and five unique MTAs in 2016 and eight unique MTAs in 2017 under LP in soil experiment. This might be due to the genotype × environment (G × E) interaction because G × E interaction is known to reduce association between phenotypic and genotypic values and cause selections from one environment to perform poorly in another, thereby forcing plant breeders to examine genotypic adaptation^80^.

In conclusion, the subpopulations derived from the genetic analysis using SNP markers also showed significant association with phenotypic performance for P efficiency traits. Among the subpopulations obtained, C2 was highly diverse and showed a very strong correlation between the traits under both P levels. Nevertheless, we found a few superior accessions which were good performer under low P stress and overlapped between soil and hydroponic screening in each subpopulation. These accessions (NI 5439, IC- 534,271, TURACO, CARAZINHO, DL 784–3, and RAJ 3777) may be included in the breeding programs to develop new cultivars tolerant to low P stress. From GWAS approach, we found 12 significant MTAs controlling two-four traits. These multi-trait MTAs could be utilized in MAS to develop wheat varieties with low P stress tolerance.

## Electronic supplementary material

Below is the link to the electronic supplementary material.

Supplementary Information 1.

Supplementary Information 2.

Supplementary Information 3.

Supplementary Information 4.
