# Supplementary figures and images for: Population structure and genome-wide association studies in bread wheat for phosphorus efficiency traits using 35 K Wheat Breeder’s Affymetrix array
Source: Sci Rep. 2021 Apr 7;11:7601. doi: 10.1038/s41598-021-87182-2 (PMC8027818; doi:10.1038/s41598-021-87182-2)

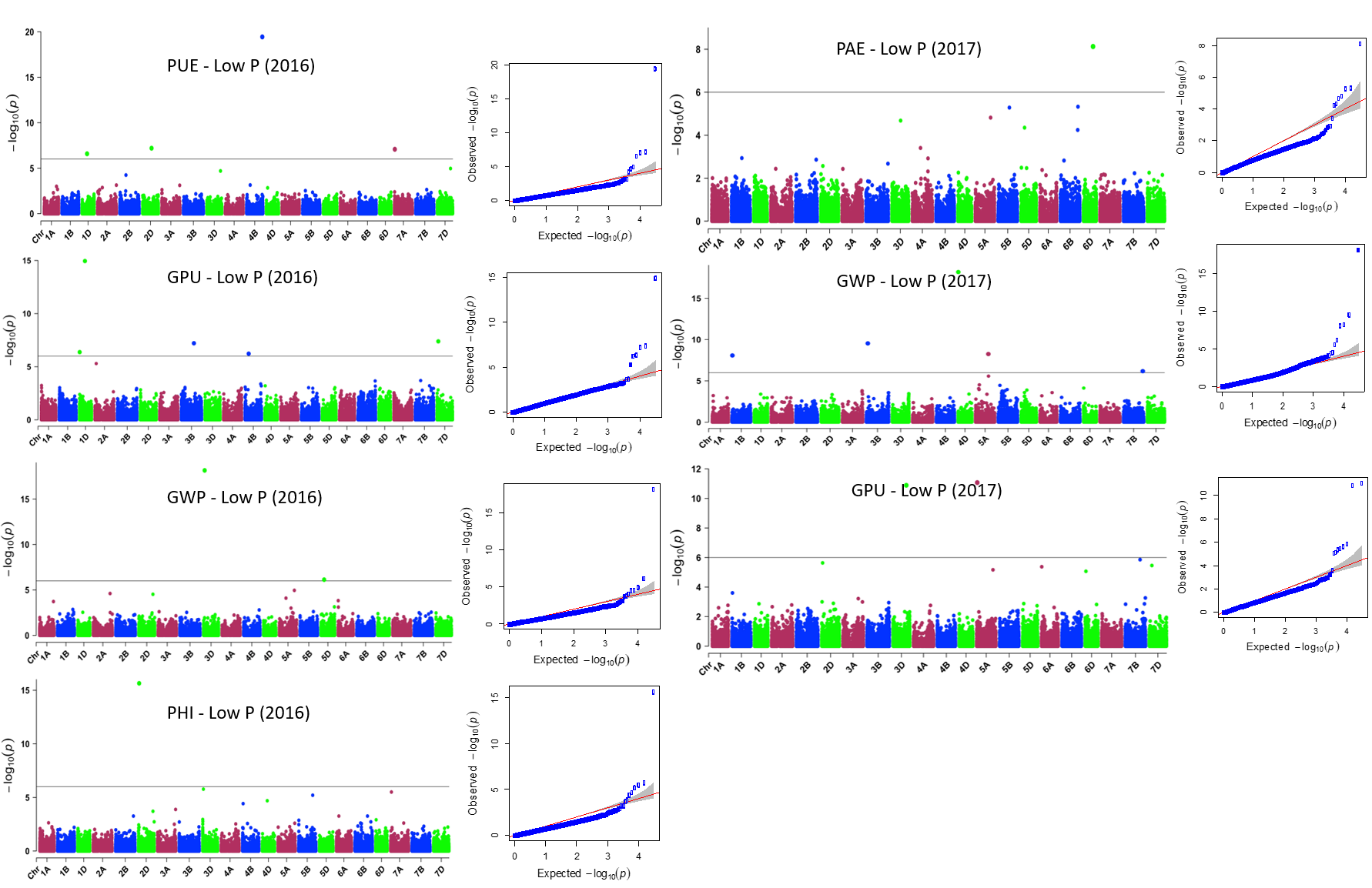

Supplement: Supplementary file 2 — Supplementary Information 2. [file 41598_2021_87182_MOESM2_ESM.tif]

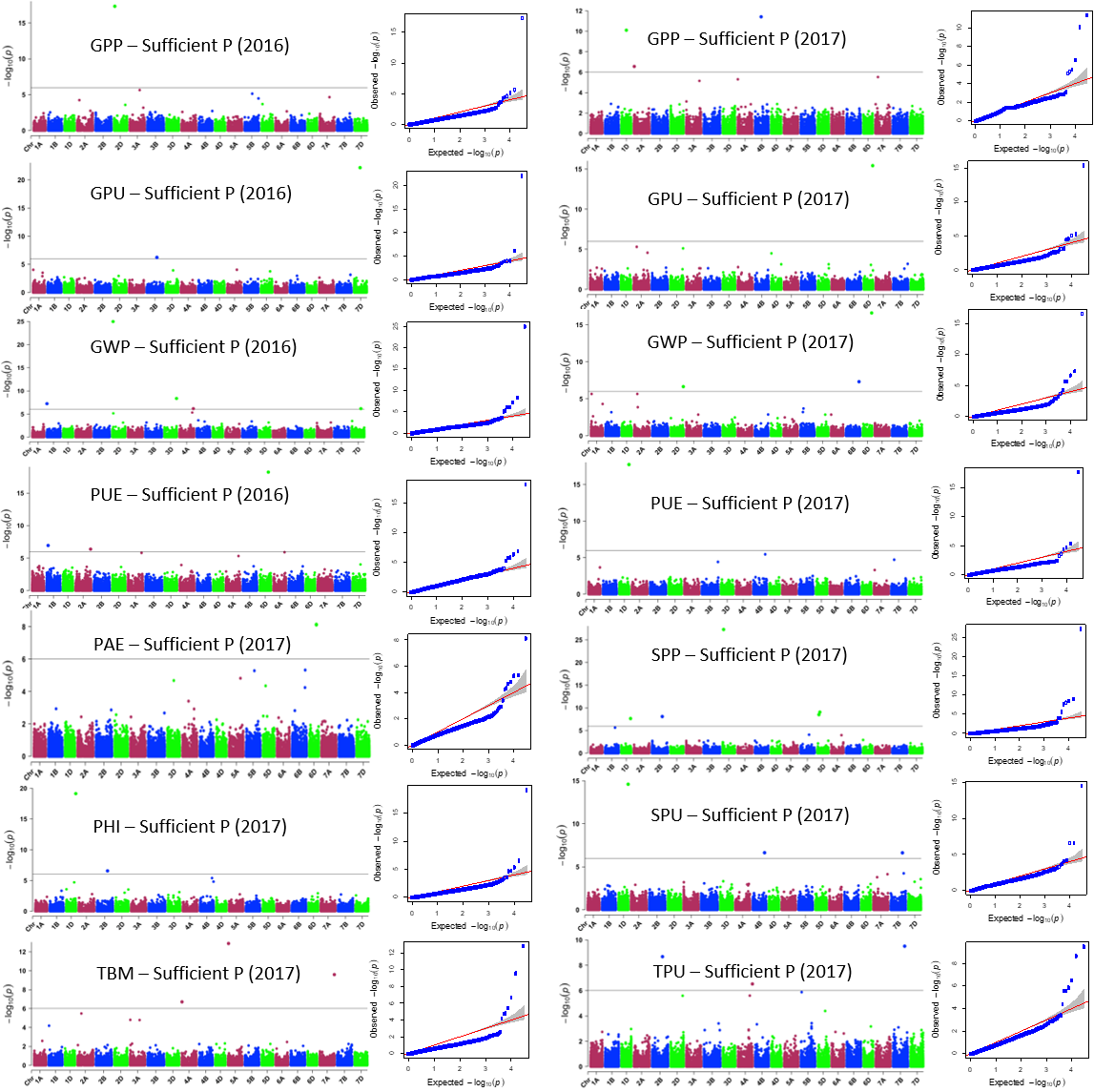

Supplement: Supplementary file 3 — Supplementary Information 3. [file 41598_2021_87182_MOESM3_ESM.tif]
