# Supplementary material for: Population structure and genome-wide association studies in bread wheat for phosphorus efficiency traits using 35 K Wheat Breeder’s Affymetrix array
Source: Sci Rep. 2021 Apr 7;11:7601. doi: 10.1038/s41598-021-87182-2 (PMC8027818; doi:10.1038/s41598-021-87182-2)
Supplement: Supplementary file 4 — Supplementary Information 4. [file 41598_2021_87182_MOESM4_ESM.docx]

*Title page:*

Population structure and genome-wide association studies in bread wheat for phosphorus efficiency traits using 35K Wheat Breeder’s Affymetrix array

Preman R. Soumya^1, #^, Amanda J. Burridge^2^, Nisha Singh^3^, Ritu Batra^1^, Renu Pandey^1^*, Sanjay Kalia^4^, Vandana Rai^3^, Keith J. Edwards^2^

^1^Mineral Nutrition Laboratory, Division of Plant Physiology, ICAR-Indian Agricultural Research Institute, New Delhi 110 012, India

^2^Life Sciences, University of Bristol, 24 Tyndall Avenue, Bristol BS8 1TQ, United Kingdom

^3^ICAR-National Institute for Plant Biotechnology, Pusa Campus, New Delhi 110 012, India

^4^Department of Biotechnology, C.G.O Complex, Lodhi Road, New Delhi-110003

^#^Present address: Regional Agricultural Research Station, Kerala Agricultural University, Ambalavayal, Wayanad 673593, Kerala, India

*****Corresponding author

Dr. Renu Pandey

Principal Scientist

Email: renu_pphy@iari.res.in

Tel.: (+91-11 25842815)

ORCID: 0000-0002-9244-8579

**Supplementary Table S1:** Pedigree information of 82 diverse accessions of bread wheat categorized into subpopulations based on population structure and diversity analysis using SNP markers. Serial number corresponds with Fig 1.

| **S. No.** | **Accession** | **Origin** | **Pedigree** |
| --- | --- | --- | --- |
|  | **SUBPOPULATION 1** | | |
| 43 | ATTILA | MEX | ND/VG 9144//KAL/BB/3/YACO/4/VEE#5 |
| 41 | BABAX | MEX | BOBWHITE/NACOZARI-76//VEERY/3/BLUEJAY/COCORAQ-75 |
| 64 | BENCUBBIN | AUS | NABAWA/GLUYAS EARLY |
| 69 | BLADE | AUS | SONORA 64//TZPP/Y54/3/KITE |
| 54 | BROOKTON | AUS | TORRES/CRANBROOK//76-W-596/CRANBROOK |
| 38 | C 306 | IND | RGN/CSK3 //2*C 591/3/C 217/N14 //C 281 |
| 39 | C 591 | IND | TYPE 9/8 B |
| 44 | CALINGIRI | AUS | CHINO/KULIN//REEVES |
| 52 | CARNAMAH | AUS | BOLSENA-1 CH/77W 660 |
| 47 | CULIACANT 89 | MEX | CM744849-2M-2Y-3M-2Y-0B-46M-0Y |
| 74 | DATAINE | AUS | HALBERD/3AG3-CONDOR*3//TINCURRIN*4 |
| 7 | DBW 16 | IND | RAJ 3765/WR 484//HUW 468 |
| 8 | DBW 88 | IND | KAUZ//ALTAR 84/AOS/3MILAN/KAUZ/4/HUITES |
| 46 | EGRET | AUS | HERON/2∗WW 15 |
| 10 | GW 322 | IND | GW 173/GW 196 |
| 82 | HD 2891 | IND | WL 711 // HD 2624 |
| 48 | MARINGA | BRA | FRONTANA/KENYA 58//PG 1 |
| 53 | NI-5439 | IND | REMP 80/3* NP 710 |
| 28 | PBW 154 | IND | HD 2160/HD 2177 |
| 72 | PBW 373 | IND | ND/VG 9144//KAL/BB/3/YCO"S'/4/VEE#5"S' |
| 29 | PBW 550 | IND | WH 594/RAJ 3858//W 485 |
| 20 | RAJ 1482 | IND | NAPO/TOB//8156/3/KAL/BB |
| 13 | RAJ 3765 | IND | HD 2402/VL639 |
| 55 | STILLETO | AUS | VERANOPOlLIS/3∗RAC177//3∗SPEAR/3/DAGGER |
| 70 | TAMMIN | AUS | BODALLIN/ERADU SIB. XBVT 223//ATLAS 66/2*MADDEN |
| 58 | VEE/MYNA | MEX | NA |
| 65 | WARIGAL | AUS | WW 15/RAVEN |
|  | **SUBPOPULATION 2** | | |
| 78 | EC-576621 |  | NA |
| 77 | GUTHA | AUS | GAMENYA//3∗GABO/KHAPSTEIN/3/3∗ FALCON/CHILE1B |
| 79 | IC-534271 |  | NA |
| 63 | OLYMPIC | AUS | BALDMIN/QUADRAT |
| 16 | SAMNGP 402 |  | NA |
| 17 | SAMNGP 404 |  | NA |
| 18 | SAMNGP 407 |  | NA |
| 66 | STRETTON | AUS | TPZ 3488 (AUS 18446)/BODALLIN |
| 50 | TURACO | MEX | CIANO-79*2/PARULA |
|  | **SUBPOPULATION 3** | | |
| 51 | ARRINO | AUS | 77-W-660/ERADU |
| 75 | BANKS | AUS | PWTH/CONDOR S’//2∗CONDOR |
| 40 | BWL 5200 | IND | EXCALIBUR/3*HD 2967 |
| 37 | BWL 5202 | IND | EXCALIBUR/3*HD 2967 |
| 62 | CARAZINHO | BRA | COLONISTA/FRONTANA |
| 1 | DBW 107 | IND | TUKURU/INQALAB91 |
| 9 | DL 784-3 | IND | KAL*4/TR 380 .27*4/3 AG/3/HD 2281 |
| 25 | DPW 621-50 | IND | KAUZ//ALTAR84/AOS/3/MILAN/KAUZ/4/HU ITES |
| 80 | EC-463394 |  | NA |
| 56 | EC-556434 |  | NA |
| 81 | EC-574376 |  | NA |
| 68 | EXCALIBUR | AUS | RAC 177/UNICULM 492//RAC 311S |
| 23 | HD 2329 | IND | SL SIB/NP 852/4/PJ SIB/P14//KT 54B/3/K 65/5/SKA/6/UP 262 |
| 2 | HD 2643 | IND | VEE'S'/HD 2407/HD 2329 |
| 24 | HD 2687 | IND | CPAN 2009/ HD 2329 |
| 11 | HD 2967 | IND | ALD/COC/URES/HD 2160M/HD 2278 |
| 4 | HD 3086 | IND | DBW 14/HD 2733//HUW 468 |
| 3 | HD 3090 | IND | SFW-16/VAISHALI/UP 2425 |
| 59 | JANZ | AUS | 3AG/4*CONDOR//COOK |
| 12 | K 9107 | IND | K 8101/K 68 |
| 45 | KATER -1 | MEX | COOK/VEE//DOVE/SERI/3/BIY/COC |
| 21 | MACS 6222 | IND | HD 2189*2//MACS 2496 |
| 57 | PBN-4364 | IND | NA |
| 35 | PBW 175 | IND | HD 2160/4/JN/GAGE//JN/KAL/3/PV18/C 273 |
| 71 | PBW 226 | IND | C 591/RN/JN/3/CHR/HIRA |
| 5 | PBW 343 | IND | ND/VG 9144//KAL/BB/3/YCO"S'/4/VEE#S "S" |
| 73 | PBW 396 | IND | CNO 67/MFD//MON'S'/3/SERI |
| 27 | PBW 502 | IND | W 485/PBW 343//RAJ 1482 |
| 26 | PBW 590 | IND | WH 594/RAJ 3814//W 485 |
| 33 | PBW 644 | IND | PBW 175/HD 2643 |
| 32 | PBW 677 | IND | PFAU/MILAN/5/CHEN/*A.squasa*//BCN/3/VEE#7/BOW/4/PAST |
| 36 | PBW 723 | IND | PBW 343+Lr57/Yr40+Lr37/Yr17 |
| 31 | PBW 725 | IND | PBW 621//GLUPRO/3*PBW 568/3/ PBW 621 |
| 34 | PBW 758 | IND | HD 3027/NW 4079 |
| 22 | RAJ 3077 | IND | HD 2267/RAJ 1482/5/BB/INIA66'S'/NAPO |
| 14 | RAJ 3777 | IND | RAJ 3160/HD 2449 |
| 49 | ROLLER-1 | MEX | FUFAN-17/VEERY-5 |
| 15 | SAMNGP 401 |  | NA |
| 19 | SAMNGP 408 |  | NA |
| 76 | SERI H-82 | MEX | KVZ/BUHO//KAL/BB |
| 67 | SILVERSTAR | AUS | PAVON S/TM 56 |
| 42 | SUNCO | AUS | SUN 9E-27∗4//3AG14//WW15/3/3∗COOK |
| 60 | SUNMIST | AUS | MISKLE SELECTION |
| 61 | SUNSTATE | AUS | HARTOG*4//COOK*5/VPM1 |
| 6 | WH 1105 | IND | MILAN/S 87230//BABAX |
| 30 | WH 416 | IND | WH 147/UP 368 |

AUS, Australia; BRA, Brazil; IND, India; MEX, Mexico; NA, Not available

**Supplementary Table S2.** Distribution of effective high-quality SNPs across all chromosomes in the A, B and D genomes of wheat.

| Chromosomes | Wheat genome | | | Total |
| --- | --- | --- | --- | --- |
|  | A | B | D |  |
| 1 | 541 | 736 | 591 | 1868 |
| 2 | 571 | 660 | 718 | 1949 |
| 3 | 459 | 530 | 402 | 1391 |
| 4 | 320 | 327 | 151 | 798 |
| 5 | 470 | 553 | 371 | 1394 |
| 6 | 380 | 527 | 321 | 1228 |
| 7 | 530 | 490 | 371 | 1391 |
| Total | 3271 | 3823 | 2925 | 10019 |

**Supplementary Table S3.** Mean value of different parameters containing number of different alleles (A), number of effective alleles (Ae), Shannon’s index (*I*), diversity index (*h*) and unbiased diversity index (uh), percentage of polymorphic loci (PPL), and number of private alleles in each of the three subpopulations comprising of 82 accessions.

| Subpopulations | A | Ae | *I* | *h* | uh | PPL (%) | No. of private alleles |
| --- | --- | --- | --- | --- | --- | --- | --- |
| C1 | 2.091 | 1.504 | 0.453 | 0.280 | 0.292 | 86.67 | 0.071 |
| C2 | 1.981 | 1.665 | 0.530 | 0.349 | 0.395 | 82.30 | 0.116 |
| C3 | 2.512 | 1.540 | 0.526 | 0.310 | 0.318 | 96.73 | 0.168 |
| Mean | 2.195 | 1.570 | 0.503 | 0.313 | 0.335 | 88.60 | 0.118 |

**Supplementary Table S4.** Square of loading values obtained from principal component analysis for different traits in 82 wheat accessions grown under sufficient (SP, 42.2 mg P kg^-1^ soil) and low (LP, 2.67 mg P kg^-1^ soil) phosphorus soil. PC1 and PC2 represents principal component 1 and 2, respectively.

| Traits | Sufficient P | | | Low P | | |
| --- | --- | --- | --- | --- | --- | --- |
|  | PC1 | PC2 | Total | PC1 | PC2 | Total |
| Total biomass | 0.003 | 0.289 | 0.292 | 0.064 | 0.103 | 0.167 |
| Grain weight per plant | 0.104 | 0.147 | 0.251 | 0.201 | 0.000 | 0.201 |
| Shoot P percentage | 0.142 | 0.000 | 0.142 | 0.028 | 0.128 | 0.156 |
| Shoot P uptake | 0.147 | 0.058 | 0.205 | 0.007 | 0.274 | 0.282 |
| Grain P percentage | 0.044 | 0.096 | 0.140 | 0.001 | 0.015 | 0.016 |
| Grain P uptake | 0.092 | 0.084 | 0.176 | 0.196 | 0.002 | 0.198 |
| Total P uptake | 0.051 | 0.226 | 0.277 | 0.116 | 0.128 | 0.244 |
| P harvest index | 0.163 | 0.011 | 0.173 | 0.106 | 0.131 | 0.237 |
| P acquisition efficiency | 0.073 | 0.052 | 0.125 | 0.105 | 0.045 | 0.150 |
| P use efficiency | 0.176 | 0.000 | 0.177 | 0.072 | 0.159 | 0.231 |
| P stress susceptibility index | 0.005 | 0.036 | 0.041 | 0.104 | 0.015 | 0.119 |

**Supplementary Table S5.** Mean, ANOVA, genetic variance (GV) and broad sense heritability (*H^2^*) for P use efficiency related traits recorded in 82 bread wheat accessions, grown under sufficient (SP, 42.2 mg P kg^-1^ soil) and low (LP, 2.67 mg P kg^-1^ soil) phosphorus soil. The pooled data over two years (2016 and 2017) was used for analysis. Abbreviations: TBM, total biomass plant^-1^; GWP, grain weight plant^-1^; SPP, shoot P percentage; SPU, shoot P uptake; GPP, grain P percentage; GPU, grain P uptake; TPU, total P uptake; PHI, P harvest index; PUE, P use efficiency. * *P*<.05; ** *P*<.01, *** *P*<.001; n.s., non-significant; P – phosphorus; G – accession; PXG – interaction between P and G.

| Traits | Mean | | *F*- value | | | GV | *H^2^*(%) |
| --- | --- | --- | --- | --- | --- | --- | --- |
|  | SP | LP | P | G | PXG |  |  |
| TBM | 27.59 | 22.13 | 3084.86*** | 48.01*** | 8.98*** | 18.67 | 94.92 |
| GWP | 12.42 | 9.46 | 3033.45*** | 47.18*** | 7.23*** | 5.43 | 93.14 |
| SPP | 0.240 | 0.110 | 9467.01*** | 27.62*** | 18.10*** | 0.00 | 91.04 |
| SPU | 37.32 | 13.94 | 8458.47*** | 33.01*** | 15.45*** | 84.67 | 91.37 |
| GPP | 0.380 | 0.310 | 3468.15*** | 20.09*** | 8.93*** | 0.00 | 84.21 |
| GPU | 46.93 | 29.25 | 6203.86*** | 30.66*** | 5.41*** | 61.33 | 91.09 |
| TPU | 84.25 | 43.19 | 14227.72*** | 16.56*** | 9.78*** | 75.33 | 83.39 |
| PHI | 57.31 | 67.62 | 1509.46*** | 44.12*** | 6.75*** | 124.33 | 93.25 |
| PUE | 15.14 | 22.01 | 6255.59*** | 40.55*** | 7.97*** | 12.33 | 92.50 |

**Supplementary Table S6.** Mean values and ANOVA of phenotypic traits of 82 wheat accessions grouped into subpopulations C1, C2, and C3 based on genotypic diversity analysis, to different levels of soil phosphorus (sufficient, 42.2 mg P kg^-1^ soil, and low, 2.67 mg P kg^-1^ soil) in the year **2016**.

| Traits | C1 | | *F*- value | | | C2 | | *F*- value | | | C3 | | *F*- value | | |
| --- | --- | --- | --- | --- | --- | --- | --- | --- | --- | --- | --- | --- | --- | --- | --- |
|  | SP | LP | P | G | PXG | SP | LP | P | G | PXG | SP | LP | P | G | PXG |
| TBM | 26.44 | 21.05 | 464.58*** | 29.39*** | 6.22*** | 24.48 | 17.51 | 200.05*** | 23.20*** | 8.60*** | 24.10 | 19.52 | 680.76*** | 35.03*** | 3.98*** |
| GWP | 11.88 | 8.21 | 648.02*** | 4.61*** | 3.55*** | 9.16 | 6.17 | 212.28*** | 72.84*** | 12.45*** | 11.24 | 8.38 | 799.30*** | 20.69*** | 3.25*** |
| SPP | 0.21 | 0.12 | 716.64*** | 14.45*** | 7.85*** | 0.21 | 0.14 | 153.95*** | 35.83*** | 13.57*** | 0.21 | 0.13 | 1024.73*** | 23.88*** | 15.93*** |
| SPU | 30.97 | 15.07 | 591.99*** | 24.85*** | 9.53*** | 33.23 | 15.76 | 139.43*** | 22.97*** | 9.64*** | 26.32 | 14.00 | 743.04*** | 13.11*** | 9.02*** |
| GPP | 0.37 | 0.34 | 104.32*** | 16.38*** | 9.76*** | 0.41 | 0.31 | 385.12*** | 66.41*** | 28.24*** | 0.40 | 0.33 | 571.17*** | 19.02*** | 5.48*** |
| GPU | 44.13 | 28.07 | 657.30*** | 5.25*** | 3.07*** | 35.08 | 18.69 | 422.30*** | 29.09*** | 13.16*** | 44.13 | 28.03 | 1271.64*** | 15.92*** | 2.82*** |
| TPU | 75.09 | 43.14 | 1400.34*** | 12.66*** | 5.63*** | 68.31 | 34.46 | 548.96*** | 11.72*** | 16.28*** | 70.45 | 42.03 | 2444.74*** | 18.12*** | 8.52*** |
| PHI | 60.22 | 65.71 | 53.94*** | 22.56*** | 5.46*** | 53.36 | 55.49 | 1.40n.s. | 31.57*** | 2.06n.s. | 63.40 | 66.46 | 23.51*** | 11.94*** | 3.82*** |
| PUE | 16.17 | 19.32 | 206.18*** | 15.35*** | 6.42*** | 13.98 | 18.70 | 80.85*** | 55.33*** | 9.89*** | 16.19 | 20.17 | 438.36*** | 15.92*** | 5.89*** |

**Supplementary Table S7.** Mean values and ANOVA of phenotypic traits of 82 wheat accessions grouped into subpopulations C1, C2, and C3 based on genotypic diversity analysis to different levels of soil phosphorus (sufficient, 42.2 mg P kg^-1^ soil, and low, 2.67 mg P kg^-1^ soil) in the year **2017**.

| Traits | C1 | | *F*- value | | | C2 | | *F*- value | | | C3 | | *F*- value | | |
| --- | --- | --- | --- | --- | --- | --- | --- | --- | --- | --- | --- | --- | --- | --- | --- |
|  | SP | LP | P | G | PXG | SP | LP | P | G | PXG | SP | LP | P | G | PXG |
| TBM | 31.36 | 26.10 | 442.62*** | 16.61*** | 6.35*** | 28.94 | 21.90 | 233.21*** | 37.18*** | 7.94*** | 29.94 | 23.97 | 1105.33*** | 34.93*** | 11.16*** |
| GWP | 13.95 | 11.37 | 328.58*** | 11.95*** | 4.86*** | 11.18 | 8.50 | 178.12*** | 103.17*** | 15.39*** | 13.89 | 10.99 | 769.77*** | 30.43*** | 7.63*** |
| SPP | 0.26 | 0.10 | 2249.19*** | 15.27*** | 12.55*** | 0.38 | 0.10 | 2284.58*** | 65.86*** | 50.32*** | 0.27 | 0.09 | 5370.32*** | 24.97*** | 19.23*** |
| SPU | 44.76 | 15.15 | 2120.04*** | 20.72*** | 12.01*** | 66.15 | 13.26 | 1459.10*** | 29.96*** | 17.79*** | 43.01 | 12.27 | 4450.41*** | 24.87*** | 14.81*** |
| GPP | 0.37 | 0.29 | 784.91*** | 5.55*** | 4.11*** | 0.39 | 0.29 | 244.05*** | 6.85*** | 2.45* | 0.37 | 0.29 | 1816.04*** | 7.85*** | 4.63*** |
| GPU | 51.94 | 32.61 | 1067.34*** | 7.89*** | 2.95*** | 42.67 | 24.92 | 522.88*** | 67.79*** | 12.48*** | 51.79 | 32.12 | 2082.90*** | 18.77*** | 6.12*** |
| TPU | 96.70 | 47.76 | 3009.63*** | 12.72*** | 6.82*** | 108.83 | 38.17 | 1959.84*** | 12.37*** | 18.35*** | 94.80 | 44.39 | 6025.58*** | 15.45*** | 8.93*** |
| PHI | 54.47 | 68.69 | 702.24*** | 23.02*** | 11.32*** | 40.16 | 64.32 | 792.08*** | 91.55*** | 8.53*** | 55.29 | 72.30 | 1719.40*** | 27.33*** | 9.59*** |
| PUE | 14.64 | 24.13 | 2006.33*** | 21.39*** | 10.10*** | 10.53 | 22.02 | 991.80*** | 51.51*** | 5.68*** | 14.84 | 24.84 | 5400.96*** | 27.88*** | 8.83*** |

**Supplementary Table S8 A**. Descriptive statistical results for P use efficiency related traits in C1 subpopulation of bread wheat grown under sufficient (42.2 mg P kg^-1^ soil) and low (2.67 mg P kg^-1^ soil) phosphorus soil. The pooled data over two years (2016 and 2017) was used for analysis. Abbreviations: TBM, total biomass plant^-1^; GWP, grain weight plant^-1^; SPP, shoot P percentage; SPU, shoot P uptake; GPP, grain P percentage; GPU, grain P uptake; TPU, total P uptake; PHI, P harvest index; PUE, P use efficiency; SD, standard deviation; CV, coefficient of variation.

| Traits | Sufficient P | | | | Low P | | | |
| --- | --- | --- | --- | --- | --- | --- | --- | --- |
|  | Mean | Range (Min-Max) | SD | CV (%) | Mean | Range (Min-Max) | SD | CV (%) |
| TBM | 28.90 | 21.91-34.93 | 3.03 | 10.49 | 23.58 | 18.08-31.97 | 3.25 | 13.76 |
| GWP | 12.91 | 10.73-14.61 | 0.99 | 7.65 | 9.79 | 7.48-11.86 | 0.93 | 9.48 |
| SPP | 0.23 | 0.14-0.31 | 0.04 | 15.98 | 0.11 | 0.08-0.15 | 0.02 | 15.06 |
| SPU | 37.87 | 15.13-57.51 | 9.98 | 26.36 | 15.11 | 8.82-26.38 | 4.53 | 29.95 |
| GPP | 0.37 | 0.35-0.45 | 0.02 | 6.01 | 0.32 | 0.25-0.36 | 0.03 | 8.43 |
| GPU | 48.03 | 38.19-56.34 | 4.62 | 9.63 | 30.34 | 24.58-38.15 | 3.20 | 10.54 |
| TPU | 85.90 | 64.19-108.08 | 9.34 | 10.88 | 45.45 | 35.54-58.90 | 5.36 | 11.79 |
| PHI | 57.35 | 41.54-78.66 | 8.09 | 14.11 | 67.20 | 53.88-76.19 | 6.99 | 10.40 |
| PUE | 15.40 | 11.36-19.89 | 1.87 | 12.13 | 21.72 | 17.46-26.68 | 2.70 | 12.41 |

**Supplementary Table S8 B**. Descriptive statistical results for P use efficiency related traits in C2 subpopulation of bread wheat grown under sufficient (42.2 mg P kg^-1^ soil) and low (2.67 mg P kg^-1^ soil) phosphorus soil.

| Traits | Sufficient P | | | | Low P | | | |
| --- | --- | --- | --- | --- | --- | --- | --- | --- |
|  | Mean | Range (Min-Max) | SD | CV (%) | Mean | Range (Min-Max) | SD | CV (%) |
| TBM | 26.71 | 18.31-31.17 | 4.41 | 16.51 | 19.71 | 14.18-23.55 | 3.37 | 17.12 |
| GWP | 10.17 | 4.70-14.42 | 3.35 | 32.93 | 7.33 | 3.42-10.62 | 2.32 | 31.67 |
| SPP | 0.30 | 0.16-0.45 | 0.10 | 32.52 | 0.12 | 0.08-0.16 | 0.02 | 21.13 |
| SPU | 49.69 | 24.97-72.33 | 15.01 | 30.20 | 14.51 | 9.00-22.94 | 4.79 | 33.03 |
| GPP | 0.40 | 0.34-0.50 | 0.06 | 15.39 | 0.30 | 0.27-0.36 | 0.03 | 9.67 |
| GPU | 38.88 | 22.49-52.41 | 9.76 | 25.10 | 21.80 | 10.96-30.68 | 6.15 | 28.22 |
| TPU | 88.57 | 68.05-108.25 | 12.65 | 14.28 | 36.31 | 28.89-41.99 | 4.80 | 13.21 |
| PHI | 46.76 | 25.35-64.01 | 12.37 | 26.46 | 59.90 | 32.76-76.20 | 13.04 | 21.77 |
| PUE | 12.26 | 5.09-16.82 | 4.24 | 34.57 | 20.36 | 10.26-26.42 | 5.31 | 26.09 |

**Supplementary Table S8 C**. Descriptive statistical results for P use efficiency related traits in C3 subpopulation of bread wheat grown under sufficient (42.2 mg P kg^-1^ soil) and low (2.67 mg P kg^-1^ soil) phosphorus soil.

| Traits | Sufficient P | | | | Low P | | | |
| --- | --- | --- | --- | --- | --- | --- | --- | --- |
|  | Mean | Range (Min-Max) | SD | CV (%) | Mean | Range (Min-Max) | SD | CV (%) |
| TBM | 26.99 | 17.53-33.62 | 3.14 | 11.62 | 21.75 | 13.64-30.06 | 3.13 | 14.41 |
| GWP | 12.56 | 5.25-14.78 | 1.56 | 12.44 | 9.68 | 4.17-12.06 | 1.70 | 17.57 |
| SPP | 0.24 | 0.17-0.42 | 0.05 | 21.10 | 0.11 | 0.07-0.15 | 0.02 | 15.90 |
| SPU | 34.58 | 21.66-56.69 | 8.20 | 23.71 | 13.13 | 8.26-21.35 | 3.23 | 24.62 |
| GPP | 0.38 | 0.34-0.47 | 0.03 | 6.53 | 0.31 | 0.26-0.36 | 0.03 | 8.00 |
| GPU | 47.86 | 23.73-56.87 | 5.70 | 11.90 | 30.07 | 14.05-39.32 | 5.71 | 19.00 |
| TPU | 82.44 | 60.36-102.60 | 8.33 | 10.11 | 43.21 | 24.26-56.84 | 6.34 | 14.67 |
| PHI | 59.36 | 34.48-70.26 | 7.04 | 11.86 | 69.38 | 48.14-81.97 | 7.05 | 10.16 |
| PUE | 15.55 | 7.16-18.52 | 1.97 | 12.67 | 22.51 | 14.41-27.41 | 2.45 | 10.88 |

**Supplementary Table S9.** Mean, ANOVA, genetic variance (GV) and broad sense heritability (*H^2^*) for P use efficiency related traits recorded in 82 bread wheat accessions, grown under sufficient (SP, 500 µM) and low (LP, 5 µM) concentration in hydroponics. Abbreviations: TBM, total biomass; SDW, shoot dry weight; RDW, root dry weight; RSR, root-to-shoot ratio; PCON, phosphorus concentration; TPU, total phosphorus uptake. **P*<.05; ** *P*<.01, *** *P*<.001; P – phosphorus; G – accession; PXG – interaction between P and G.

| Traits | Mean | | *F*- value | | | GV | *H^2^*(%) |
| --- | --- | --- | --- | --- | --- | --- | --- |
|  | SP | LP | P | G | PXG |  |  |
| TBM | 0.279 | 0.235 | 467.23*** | 69.69*** | 5.54*** | 0.01 | 94.46 |
| SDW | 0.244 | 0.191 | 752.17*** | 52.93*** | 5.48*** | 0.01 | 93.18 |
| RDW | 0.034 | 0.044 | 1056.07*** | 82.60*** | 10.76*** | 0.00 | 95.39 |
| RSR | 0.138 | 0.240 | 1566.17*** | 11.16*** | 9.64*** | 0.00 | 72.15 |
| PCON | 0.471 | 0.148 | 59788.24*** | 19.66*** | 18.84*** | 0.00 | 100.00 |
| TPU | 1.305 | 0.342 | 13379.58*** | 41.58*** | 22.84*** | 0.12 | 92.00 |

**Supplementary Table S10 A**. Descriptive statistical results for P use efficiency related traits in C1 subpopulation of bread wheat grown under sufficient P (500 µM) and low P (5 µM) concentration in hydroponics. Abbreviations: TBM, total biomass; SDW, shoot dry weight; RDW, root dry weight; RSR, root-to-shoot ratio; PCON, phosphorus concentration; TPU, total phosphorus uptake; CV, coefficient of variation; SD, standard deviation.

| Traits | Sufficient P | | | | Low P | | | |
| --- | --- | --- | --- | --- | --- | --- | --- | --- |
|  | Mean | Range (Min-Max) | SD | CV (%) | Mean | Range (Min-Max) | SD | CV (%) |
| TBM | 0.297 | 0.170-0.520 | 0.098 | 32.89 | 0.249 | 0.100-0.430 | 0.080 | 32.07 |
| SDW | 0.260 | 0.148-0.456 | 0.082 | 31.62 | 0.204 | 0.075-0.363 | 0.067 | 32.75 |
| RDW | 0.037 | 0.013-0.072 | 0.016 | 44.09 | 0.045 | 0.017-0.081 | 0.017 | 36.94 |
| RSR | 0.138 | 0.080-0.180 | 0.027 | 19.78 | 0.229 | 0.110-0.490 | 0.075 | 32.70 |
| PCON | 0.469 | 0.414-0.604 | 0.036 | 7.61 | 0.151 | 0.091-0.212 | 0.021 | 14.09 |
| TPU | 1.406 | 0.763-2.428 | 0.436 | 31.01 | 0.345 | 0.175-0.572 | 0.107 | 31.15 |

**Supplementary Table S10 B**. Descriptive statistical results for P use efficiency related traits in C2 subpopulation of bread wheat grown under sufficient P (500 µM) and low P (5 µM) concentration in hydroponics.

| Traits | Sufficient P | | | | Low P | | | |
| --- | --- | --- | --- | --- | --- | --- | --- | --- |
|  | Mean | Range (Min-Max) | SD | CV (%) | Mean | Range (Min-Max) | SD | CV (%) |
| TBM | 0.262 | 0.140-0.540 | 0.113 | 43.09 | 0.197 | 0.110-0.370 | 0.082 | 41.70 |
| SDW | 0.232 | 0.110-0.483 | 0.104 | 44.97 | 0.157 | 0.081-0.307 | 0.070 | 44.75 |
| RDW | 0.029 | 0.019-0.061 | 0.013 | 44.33 | 0.041 | 0.028-0.067 | 0.013 | 30.98 |
| RSR | 0.130 | 0.080-0.230 | 0.044 | 34.19 | 0.278 | 0.200-0.400 | 0.073 | 26.26 |
| PCON | 0.489 | 0.374-0.583 | 0.061 | 12.41 | 0.124 | 0.093-0.161 | 0.024 | 19.31 |
| TPU | 1.193 | 0.652-2.688 | 0.592 | 49.63 | 0.239 | 0.157-0.372 | 0.072 | 29.95 |

**Supplementary Table S10 C.** Descriptive statistical results for P use efficiency related traits in C3 subpopulation of bread wheat grown under sufficient P (500 µM) and low P (5 µM) concentration in hydroponics.

| Traits | Sufficient P | | | | Low P | | | |
| --- | --- | --- | --- | --- | --- | --- | --- | --- |
|  | Mean | Range (Min-Max) | SD | CV (%) | Mean | Range (Min-Max) | SD | CV (%) |
| TBM | 0.272 | 0.160-0.430 | 0.074 | 27.27 | 0.234 | 0.120-0.360 | 0.061 | 26.03 |
| SDW | 0.238 | 0.133-0.387 | 0.064 | 27.12 | 0.189 | 0.088-0.294 | 0.051 | 26.90 |
| RDW | 0.033 | 0.010-0.061 | 0.011 | 34.39 | 0.044 | 0.027-0.075 | 0.013 | 29.42 |
| RSR | 0.140 | 0.060-0.240 | 0.033 | 23.46 | 0.240 | 0.150-0.390 | 0.060 | 24.91 |
| PCON | 0.462 | 0.298-0.599 | 0.047 | 10.25 | 0.145 | 0.079-0.183 | 0.026 | 18.02 |
| TPU | 1.267 | 0.719-2.164 | 0.358 | 28.22 | 0.360 | 0.181-0.557 | 0.097 | 26.88 |

**Table S11.** List of significant SNPs with their position on the chromosome and p-value associated with traits measured under soil at different phosphorus (P) levels. Abbreviations: TBM, total biomass plant^-1^; GWP, grain weight plant^-1^; SPP, shoot P percentage; SPU, shoot P uptake; GPP, grain P percentage; GPU, grain P uptake; TPU, total P uptake; PHI, P harvest index; PUE, P use efficiency; PAE, P acquisition efficiency.

| P level | Trait | Year | Marker | Chromosome | Position (bp) | p-value | SNP Type | Consequence Type |
| --- | --- | --- | --- | --- | --- | --- | --- | --- |
| Sufficient P | GPP | 2016, Pooled | AX-94481552 | 2D | 36798253 | 4.72E-18 | M | synonymous |
|  |  | 2017 | AX-95163344 | 4B | 302868468 | 3.89E-12 | Y | 3' UTR |
|  |  | 2017 | AX-94739727 | 1D | 250140206 | 7.97E-11 | M | stop gained |
|  |  | 2017 | AX-95003568 | 2A | 17001297 | 2.79E-07 | S | synonymous |
|  | GPU | 2016 | AX-94506335 | 7D | 299594792 | 6.72E-23 | Y | missense |
|  |  | 2016 | AX-95023178 | 3B | 515148935 | 5.89E-07 | Y | intergenic |
|  |  | 2017 | AX-94585983 | 6D | 451762695 | 3.71E-16 | R | missense |
|  |  | Pooled | AX-95087092 | 1D | 484402795 | 8.55E-21 | Y | synonymous |
|  | GWP | 2016 | AX-94481552 | 2D | 36798253 | 1.06E-25 | M | synonymous |
|  |  | 2016 | AX-95167695 | 3D | 610354329 | 4.36E-09 | Y | missense |
|  |  | 2016 | AX-94955419 | 1B | 4478899 | 5.87E-08 | M | 3' UTR |
|  |  | 2016 | AX-94642006 | 4A | 708650418 | 6.90E-07 | S | missense |
|  |  | 2016 | AX-95257093 | 7D | 464947781 | 6.91E-07 | K | synonymous |
|  |  | 2017 | AX-94585983 | 6D | 451762695 | 2.50E-17 | R | missense |
|  |  | 2017 | AX-94453018 | 6B | 669014108 | 4.84E-08 | R | intergenic |
|  |  | 2017 | AX-95003018 | 2D | 639678198 | 2.26E-07 | S | synonymous |
|  |  | Pooled | AX-95210917 | 2B | 64994486 | 6.75E-16 | Y | synonymous |
|  | PUE | 2016 | AX-94391831 | 5D | 439786337 | 6.05E-19 | Y | intergenic |
|  |  | 2016 | AX-94761160 | 1B | 72980531 | 1.13E-07 | R | intergenic |
|  |  | 2016 | AX-94472538 | 2A | 758692139 | 4.15E-07 | K | intergenic |
|  |  | 2016 | AX-94468687 | 6A | 554104441 | 1.23E-06 | W | 3' UTR |
|  |  | 2016 | AX-94396499 | 3A | 685357955 | 1.58E-06 | M | missense |
|  |  | 2017 | AX-95087092 | 1D | 484402795 | 1.88E-18 | Y | synonymous |
|  |  | pooled | AX-94481552 | 2D | 36798253 | 1.12E-17 | M | synonymous |
|  |  | pooled | AX-94781104 | 3A | 556359292 | 1.17E-08 | W | missense |
|  |  | pooled | AX-94403103 | 6B | 574033949 | 8.13E-07 | Y | synonymous |
|  | PHI | 2017, Pooled | AX-95087092 | 1D | 484402795 | 7.53E-20 | Y | synonymous |
|  |  | 2017 | AX-94414210 | 2B | 534711855 | 2.74E-07 | Y | missense |
|  |  | Pooled | AX-94481552 | 2D | 36798253 | 4.93E-19 | M | synonymous |
|  |  | Pooled | AX-94641695 | 1A | 494450343 | 2.34E-10 | Y | synonymous |
|  |  | Pooled | AX-94863246 | 4A | 732512417 | 4.21E-09 | Y | synonymous |
|  |  | Pooled | AX-94434246 | 7A | 535905383 | 7.67E-07 | Y | downstream gene |
|  |  | Pooled | AX-94599600 | 6D | 3942611 | 1.05E-06 | S | intergenic |
|  |  | Pooled | AX-94858246 | 1B | 367441581 | 1.37E-06 | S | missense |
|  | PAE | 2017 | AX-95223197 | 6D | 354900155 | 7.43E-09 | Y | 3' UTR |
|  | SPP | 2017 | AX-94507987 | 3D | 44824191 | 5.27E-28 | M | 3' UTR |
|  |  | 2017 | AX-95126535 | 5D | 129865308 | 8.82E-10 | R | synonymous |
|  |  | 2017 | AX-95007273 | 5D | 83905012 | 3.19E-09 | Y | intergenic |
|  |  | 2017 | AX-94472688 | 2B | 522087336 | 8.22E-09 | R | intron |
|  |  | 2017 | AX-94728619 | 1D | 467598305 | 2.31E-08 | K | missense |
|  |  | Pooled | AX-94396214 | 1B | 292341939 | 4.79E-24 | Y | stop lost |
|  |  | Pooled | AX-95240693 | 3A | 10302316 | 1.90E-10 | R | synonymous |
|  |  | Pooled | AX-94840416 | 2B | 734127065 | 8.60E-09 | M | 3' UTR |
|  |  | Pooled | AX-94691660 | 1A | 146944613 | 2.55E-08 | M | 5' UTR |
|  |  | Pooled | AX-94546428 | 4B | 481576195 | 4.91E-08 | Y | intergenic |
|  | SPU | 2017 | AX-95087092 | 1D | 484402795 | 2.55E-15 | Y | synonymous |
|  |  | 2017 | AX-94528470 | 4B | 595269330 | 2.31E-07 | M | intergenic |
|  |  | 2017 | AX-95085037 | 7B | 586755395 | 2.38E-07 | W | intergenic |
|  |  | Pooled | AX-94459431 | 5D | 261988499 | 4.55E-11 | R | intron |
|  | TBM | 2017 | AX-94840782 | 5A | 18797342 | 1.33E-13 | S | missense |
|  |  | 2017 | AX-94659296 | 7A | 735413194 | 2.58E-10 | S | missense |
|  |  | 2017 | AX-94797959 | 4A | 12623918 | 1.96E-07 | R | synonymous |
|  | TPU | 2017 | AX-95085037 | 7B | 586755395 | 3.12E-10 | W | intergenic |
|  |  | 2017 | AX-94472688 | 2B | 522087336 | 2.10E-09 | R | intron |
|  |  | 2017 | AX-95217431 | 4A | 733991976 | 2.98E-07 | K | missense |
|  | PSSI | Pooled | AX-94381520 | 3A | 44628840 | 6.95E-07 | Y | synonymous |
| Low P | GPU | 2016, Pooled | AX-95081619 | 7D | 52851501 | 4.12E-08 | Y | missense |
|  |  | 2016 | AX-94815193 | 1D | 224574162 | 1.13E-15 | K | missense |
|  |  | 2016 | AX-95143961 | 3B | 513665765 | 6.18E-08 | Y | missense |
|  |  | 2016 | AX-94811887 | 1D | 8465616 | 4.24E-07 | M | intergenic |
|  |  | 2016 | AX-94742877 | 4B | 149722720 | 5.95E-07 | R | synonymous |
|  |  | 2017 | AX-94840782 | 5A | 18797342 | 8.52E-12 | S | missense |
|  |  | 2017 | AX-94989529 | 3D | 487812517 | 1.30E-11 | Y | synonymous |
|  |  | pooled | AX-94507987 | 3D | 44824191 | 6.31E-18 | M | 3' UTR |
|  |  | pooled | AX-94527055 | 3B | 778277426 | 1.12E-06 | K | intergenic |
|  |  | pooled | AX-94979364 | 1A | 10972400 | 1.24E-06 | S | synonymous |
|  | GWP | 2016, Pooled | AX-94507987 | 3D | 44824191 | 7.00E-19 | M | 3' UTR |
|  |  | 2016, Pooled | AX-95126535 | 5D | 129865308 | 7.38E-07 | R | synonymous |
|  |  | 2017 | AX-94757759 | 4D | 5554992 | 6.65E-19 | S | missense |
|  |  | 2017 | AX-95154536 | 3B | 55128771 | 2.87E-10 | Y | synonymous |
|  |  | 2017 | AX-94638131 | 5A | 476439664 | 5.58E-09 | S | intergenic |
|  |  | 2017 | AX-94966346 | 1B | 11937396 | 8.36E-09 | S | intergenic |
|  |  | 2017 | AX-94677860 | 7B | 716369897 | 6.68E-07 | S | synonymous |
|  |  | pooled | AX-94697260 | 6D | 6315570 | 1.55E-11 | S | missense |
|  |  | pooled | AX-95126535 | 5D | 129865308 | 8.44E-07 | R | synonymous |
|  | PUE | 2016, Pooled | AX-94422147 | 4B | 649525711 | 3.61E-20 | Y | intergenic |
|  |  | 2016, Pooled | AX-94470776 | 2D | 369482459 | 6.36E-08 | R | synonymous |
|  |  | 2016, Pooled | AX-94557144 | 7A | 14345578 | 8.32E-08 | S | intergenic |
|  |  | 2016, Pooled | AX-94480700 | 1D | 205483669 | 2.59E-07 | S | synonymous |
|  |  | Pooled | AX-94612526 | 6A | 12287533 | 3.41E-07 | S | 3' UTR |
|  | PHI | 2016 | AX-94937541 | 2D | 32294485 | 2.25E-16 | S | missense |
|  |  | Pooled | AX-94381520 | 3A | 44628840 | 6.95E-07 | Y | synonymous |
|  |  | Pooled | AX-94873200 | 7A | 471000696 | 4.97E-05 | R | synonymous |
|  |  | Pooled | AX-94792627 | 5B | 397837909 | 8.79E-05 | Y | 3' UTR |
|  | PAE | 2017 | AX-95223197 | 6D | 354900155 | 7.43E-09 | Y | 3' UTR |
|  | PSSI | Pooled | AX-94381520 | 3A | 44628840 | 6.95E-07 | Y | synonymous |

**Table S12.** List of significant SNPs with their position on the chromosome and p-value associated with traits measured under hydroponic conditions at different phosphorus (P) levels. Abbreviations: TBM, total biomass; SDW, shoot dry weight; PCON, phosphorus concentration; TPU, total phosphorus uptake.

| P level | Trait | Year | Marker | Chromosome | Position (bp) | p-value | SNP Type | Consequence Type |
| --- | --- | --- | --- | --- | --- | --- | --- | --- |
| Sufficient P | TPU | 2016 | AX-94687416 | 2A | 692633149 | 1.15E-06 | R | Synonymous |
|  |  | 2016 | AX-95108407 | 5B | 170635919 | 7.01E-06 | R | Intergenic |
|  | SDW | 2016 | AX-94687416 | 2A | 692633149 | 2.18E-06 | R | Synonymous |
|  |  | 2016 | AX-95108407 | 5B | 170635919 | 2.06E-05 | R | Intergenic |
|  | TBM | 2016 | AX-94687416 | 2A | 692633149 | 5.00E-06 | R | Synonymous |
|  |  | 2016 | AX-94718390 | 2B | 763840182 | 3.70E-05 | Y | Intergenic |
|  |  | 2016 | AX-95108407 | 5B | 170635919 | 1.92E-05 | R | Missense |
| Low P | PCON | 2016 | AX-94998682 | 1D | 128526613 | 8.40E-08 | Y | Missense |
|  |  | 2016 | AX-95156986 | 2D | 401290110 | 3.23E-07 | R | Synonymous |
|  |  | 2016 | AX-94952598 | 5A | 168864156 | 9.67E-08 | Y | Missense |
|  |  | 2016 | AX-95180361 | 6A | 41096583 | 1.73E-06 | W | Intergenic |
|  |  | 2016 | AX-94793154 | 6D | 382352310 | 3.47E-05 | R | Intron |
|  | TBM | 2016 | AX-95108407 | 5B | 170635919 | 1.37E-05 | R | Intergenic |
|  |  | 2016 | AX-94622790 | 7B | 13827968 | 2.10E-05 | R | Intergenic |

**Supplementary Figure S1:** Weather data of the cropping duration of bread wheat grown for two seasons (A) year 2015-16, and (B) year 2016-17

**Supplementary Figure S2:**  Distribution of Polymorphic Information Content (PIC) across (A) the homoeologous group 1 to 7, and (B) the wheat genomes A, B and D.

**Supplementary Figure S3:**  Estimated population structure of 82 wheat accessions (*K* = 3) using STRUCTURE. Delta *K* (Δ*K*) for different number of subpopulations (*K*).

**Supplementary Figure S4.** Manhattan plots showing SNP markers associated with different traits and Quantile-Quantile plots for 82 bread wheat accessions grown in low phosphorus soil for years 2016 and 2017. The horizontal line represents FDR adjusted p*<* 0.001. The threshold of -log_10_ (P-value) ≥ 6.0 was used as a cutoff to identify association analysis. Abbreviations: PUE, P use efficiency; GPU, grain P uptake; GWP, grain weight per plant; PHI, P harvest index; PAE, P acquisition efficiency.

**Supplementary Figure S5.** Manhattan plots showing SNP markers associated with different traits and Quantile-Quantile plots for 82 bread wheat accessions grown in sufficient P soil for years 2016 and 2017. The horizontal line represents FDR adjusted p*<* 0.001. The threshold of -log_10_ (P-value) ≥ 6.0 was used as a cutoff to identify association analysis. Abbreviations: GPP, grain P percentage; GPU, grain P uptake; GWP, grain weight per plant; PUE, P use efficiency; PAE, P acquisition efficiency; PHI, P harvest index; TBM, total biomass per plant; SPP, shoot P percentage; SPU, Shoot P uptake; TPU, total P uptake.
